# Charge Photoaccumulation in Covalent Polymer Networks for Boosting Photocatalytic Nitrate Reduction to Ammonia

**DOI:** 10.1002/advs.202401878

**Published:** 2024-04-06

**Authors:** Xinjia He, Yingke Wen, Yanjie Fang, Mengjie Li, Bing Shan

**Affiliations:** ^1^ Department of Chemistry Key Laboratory of Excited‐State Materials of Zhejiang Province Zhejiang University Hangzhou 310058 China

**Keywords:** charge photoaccumulation, molecular assembly, organic photoelectrode, photoelectrocatalysis

## Abstract

In the design of photoelectrocatalytic cells, a key element is effective photogeneration of electron‐hole pairs to drive redox activation of catalysts. Despite recent progress in photoelectrocatalysis, experimental realization of a high‐performance photocathode for multi‐electron reduction of chemicals, such as nitrate reduction to ammonia, has remained a challenge due to difficulty in obtaining efficient electrode configurations for extraction of high‐throughput electrons from absorbed photons. This work describes a new design for catalytic photoelectrodes using chromophore assembly‐functionalized covalent networks for boosting eight‐electron reduction of nitrate to ammonia. Upon sunlight irradiation, the photoelectrode stores a mass of reducing equivalents at the photoexcited chromophore assembly for multielectron reduction of a copper catalyst, enabling efficient nitrate reduction to ammonia. By introducing the new photoelectrode structure, it is demonstrated that the electronic interplay between charge photo‐accumulating assembly and multi‐electron redox catalysts can be optimized to achieve proper balance between electron transfer dynamics and thermodynamic output of photoelectrocatalytic systems.

## Graphical abstract

A new design is presented for catalytic photoelectrodes using chromophore assembly‐functionalized covalent networks for nitrate reduction to ammonia. Upon sunlight irradiation, the photoelectrode stores a mass of reducing equivalents at the chromophore assembly for multielectron reduction of a copper catalyst, enabling efficient nitrate reduction to ammonia at quantum efficiency of 14% with photocurrent at ≈3.0 mA cm^−2^ for tens of hours.

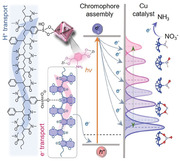

## 1 Introduction

In artificial photosystems based on photoelectrocatalytic (PEC) cells, production of solar fuels generally requires coupling of the separated electrical charges with multielectron catalysts for the half reactions of interest.^[^ ^1^, ^2^, ^3^, ^4^, ^5^, ^6^, ^7^, ^8^, ^9^ ^]^ Although PEC technique presents high potential in practical solar fuel production, photoelectrode materials for redox catalysis usually exhibit low efficiencies due to limitations in generating high density photoelectrons for catalyst activation.^[^ ^10^, ^11^ ^]^ Typical molecular photoelectrodes use single‐molecular complexes as photosensitizers that are supported on inorganic semiconductors.^[^ ^11^, ^12^, ^13^, ^14^ ^]^ For those systems, the number of photogenerated charge‐separated states is restricted by the low density of the photosensitizers. Most photoinduced redox catalysis involves multi‐electron transfer processes, while photosensitizers usually produce monoelectronic charge separation states. So far, only a few molecular photoactive systems with a designed charge accumulation site have been described. Fabrication of all‐integrated photoelectrodes that can convert sunlight energy and accumulate charges to finally use them in multielectron catalytic reactions is of high interest, but challenging due to difficulty in integration of stable photosensitizers at high loading density.

Molecular assembly could have similar photocatalytic activities as that of their metal complex ensembles, but with advantages in surface immobilization due to solid‐state nature and in high surface area that exposes abundant active sites.^[^ ^15^, ^16^, ^17^, ^18^ ^]^ Organizing photosensitizers in high‐density molecular assembly could, in principle, overcome limitations in insufficient charge‐separation states of current catalytic photoelectrodes. As chromophore assembly being integrated directly in photoelectrodes, a limitation exists in low injection efficiency due to ineffective contact between chromophore and the electrode substrate. For a typical photocathode, upon solar irradiation, each chromophore unit in the assembly is excited to generate a relatively high‐energy state which injects the photogenerated hole to a p‐type semiconducting substrate. The injection efficiency is dictated by the interfacial charge transfer dynamics between the chromophore and the semiconductor. For photoelectrodes based on typical inorganic electrode substrates, the contacts between chromophores and semiconductors are deficient and unstable, which limits the PEC performances.

In situ integration of molecular assembly in semiconducting covalent networks should be a desirable way to tackle the charge‐injection and physicochemical stability limitations. However, given the structures and formation conditions of chromophore assemblies, finding an appropriate covalent substrate for in situ assembly integration is difficult, due to the low thermal stability of many semiconducting organic polymers.^[^ ^19^, ^20^, ^21^, ^22^ ^]^ In this work, we introduce a charge‐accumulating photoelectrode whose photosensitizing component is a chromophore assembly (CA) composed of zirconium‐coordinated 2‐aminoterephthalic acid. The CA is integrated in situ in thermally stable, p‐type poly(3,4‐ethylenedioxythiophene) (PEDOT) networks. Owing to the large porosity and high thermal stability of the covalent substrate, CA can be synthesized in the photoelectrode with homogeneous distribution. This all‐integrated electrode/CA system can store large flux of reducing equivalents at the conduction band of CA upon irradiation with sunlight. The accumulated electrons at CA can be subsequently transferred to a copper catalyst, effecting efficient nitrate (NO_3_^−^) reduction to ammonia (NH_3_). The integrated photoelectrode shows greatly improved PEC activity, with a high external quantum efficiency (EQE) for solar‐driven NO_3_^−^‐to‐NH_3_ conversion at 14% and stable cathodic photocurrent density at 3.0 mA cm^−2^ for tens of hours. This work outlines the beneficial effects of charge photoaccumulation in catalytic photoelectrodes for driving multielectron transfer redox reactions.

## 2 Results and Discussion

### 2.1 Chromophore Assembly for Charge Photoaccumulation

**Figure 1:**
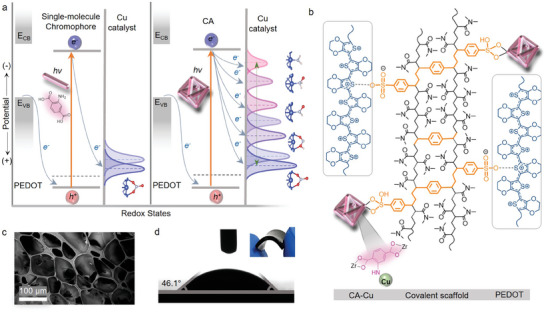
Charge photoaccumulation for activation of Cu catalyst. a) Potential diagrams showing the differences in catalyst activation by the excited states of single‐molecule chromophore (left) and chromophore assembly (CA) (right). b) Structure of the organic photoelectrode (HrHE‐CA‐Cu). c,d) Scanning electron microscopy (SEM) micrograph (c) and contact angle (d) (water drop volume: 3 µL) for HrHE‐CA‐Cu with an inset showing the optical image of the photoelectrode.

The CA photocathode is constructed by in situ integration of zirconium‐coordinated 2‐aminoterephthalic acid in a heat‐resistant hydrogel electrode (HrHE), resulting in the photoelectrode HrHE‐CA in **Figure** **1**. The design principle for the CA photoelectrode relies on photogeneration of multiple charge‐separated states at CA which delivers electrons at the conduction band to the Cu catalyst toward eight‐electron reduction of NO_3_^−^, as illustrated in Figure 1a. Compared with the photoelectrode with single‐molecular chromophore, the CA photoelectrode is able to store a mass of reducing equivalents at the photoexcited states, providing a prerequisite for successive reduction of catalytic intermediates during NO_3_^−^‐to‐NH_3_ conversion. A systematic approach for synthesizing CA photoelectrode (structure in Figure 1b) is developed here based on screening for stable covalent electrode substrates. The synthetic procedure shown in Figure S1 (Supporting Information) starts with formation of a heat‐resistant hydrogel (HrH) scaffold which is divinylbenzene (DVB)‐crosslinked copolymer of N, N‐dimethylacrylamide (DMA) and styrenesulfonate (SS). Fourier‐transform infrared spectra in Figure S2 (Supporting Information) evince the successful combination of SS and DMA in HrH. Incorporation of PEDOT in HrH to form HrHE is achieved by in situ polymerization of 3,4‐ethylenedioxythiophene after pre‐saturation of HrH with the monomer.

The strong ionic interaction between thiophene rings at PEDOT and ─SO_3_^−^ pendants at HrH enables monodispersion of PEDOT in HrHE, leading to high hole conductivity^[^ ^23^ ^]^ of the covalent substrate. The polymerization conditions, including monomer concentration and reaction time, are adjusted based on the hole‐conducting behaviors of the resulting HrHE (Figure S3, Supporting Information). From the results, polymerization of EDOT (0.50 m) for 24 h affords HrHE with optimum hole conductivity relative to the 6‐ and 12‐h polymerized samples, which is adopted as the synthetic method for the HrHE. In this method, close intermolecular interaction and packing motif of the ionically crosslinked PEDOT can be preserved at elevated temperatures (vide infra) for in situ integration of CA. For the photocathode, CA is employed as a photosensitizer given its unique advantages such as high photoactivity, porosity and large surface area.^[^ ^16^, ^24^ ^]^ As illustrated in Figure S4 (Supporting Information), integration of CA in HrHE networks as a photosensitizer is achieved using a solvothermal method with the ligand, 2‐aminoterephthalic acid, in the presence of diluted hydrochloric acid as an acidic modulator. The integrated CA disperses uniformly in HrHE by reversible coordination between Zr^IV^ nodes at CA and ─SO_3_H groups at HrHE. The X‐ray diffraction patterns (Figure S5, Supporting Information) and X‐ray photoelectron spectroscopy (XPS) spectra (Figure S6, Supporting Information) for the synthesized CA evince successful integration of it in the photocathode.

Compared with typical photoelectrodes for catalysis, the CA photoelectrodes introduced here are scalable in three dimensions with millimeter‐scale thickness, and highly flexible owing to the entirely covalent scaffold, as shown in Figure 1c and Figure S7 (Supporting Information). The photocathode also exhibits typical hydrogel features such as high hydrophilicity (contact angles in Figure 1d) that is advantageous for aqueous catalytic reactions, and micrometer‐scale pores (Figure 1c) for accommodation of monodispersing CA molecules. The resulting CA photocathode, HrHE‐CA, is functionalized with a Cu catalyst by site‐selective photodeposition of Cu at CA to serve as catalytic centers for NO_3_^−^ reduction to NH_3_. Physical properties including contact angles, average pore sizes and porosities for the final photocathode are summarized in Table S1 (Supporting Information). The light capturing property of CA in the photoelectrode is shown in Figure S8 (Supporting Information) which exhibits a broader light absorption region relative to the single‐molecule chromophore, C.

**Figure 2:**
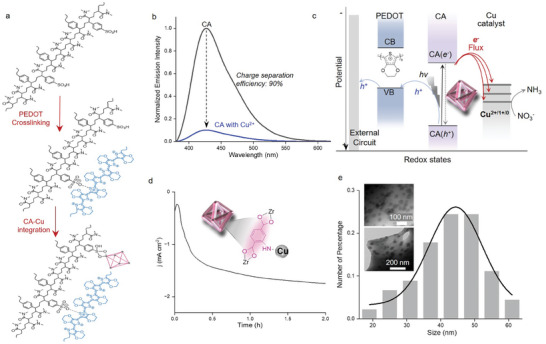
Photoinduced site‐selective integration of the Cu catalyst. a) Catalyst integration to generate the photoelectrode, HrHE‐CA‐Cu. b) Photoinduced charge separation evaluated using emission quenching of CA^*^ excited state. The emission intensity was normalized based on the absorption efficiency of CA at the excitation wavelength. c) Potential diagram illustrating the energy levels with arrows showing directions of the photoinduced charge transfer. d) Photoelectrochemical deposition of the Cu catalyst. e) Morphology and particle size distribution of the catalyst in the photoelectrode characterized by transmission electron microscopy (TEM).

The Cu catalyst is photoelectrochemically deposited at the CA sites in the photoelectrode, with details given in **Figure** **2**. Upon solar irradiation, the photoelectrode, HrHE‐CA, generates electrons carried by the CA with a reducing power of −0.37 V vs NHE (Table S2, Supporting Information). The photogenerated electrons are subsequently transferred to Cu^2+^ ions adsorbed at zirconium sites of CA, with a driving force of 0.71 eV based on E(Cu^2+/0^) (0.34 V vs NHE), leading to Cu catalyst deposition at CA. The synthetic procedure is illustrated in Figure 2a. For the CA chromophore, its excited state (CA^*^) reacts with Cu^2+^ via interfacial oxidative quenching, which leads to the decrease in emission intensity in Figure 2b. Since there is no other quenching process for CA^*^, the efficiency of photoinduced electron transfer from CA^*^ to Cu^2+^ is estimated as 90%, based on the relative photoluminescence intensities of CA^*^ in Figure 2b. The formation and transfer of photogenerated charge‐separation states is schematically illustrated in the potential diagram in Figure 2c. Photogenerated holes at the valence band of CA is quite high in energy (Table S2, Supporting Information) which can be captured by PEDOT at HrHE. The driving force for photogenerated hole transfer is as large as 3.26 eV (Table S2, Supporting Information), based on the results from Tauc and Mott–Schottky analyses of PEDOT in Figure S9 (Supporting Information).

The catalytic photoelectrode is obtained by 2‐h photoelectrochemical deposition of the Cu catalyst in HrHE‐CA, with the photocurrent densities shown in Figure 2d. SEM with elemental mapping of Cu in Figure S10 (Supporting Information) illustrates the homogeneous distribution of Cu catalyst in the photoelectrode. An average particle size of 40–50 nm can be derived from transmission electron microscopic (TEM) image with size distribution in Figure 2e. Based on the surface coverage of each component in the photoelectrode (Table S3, Supporting Information), the density of Cu is about half of the chromophore, 2‐aminoterephthalic acid, in CA. The XPS spectra in Figures S11 and S12 (Supporting Information) for HrHE‐CA before and after Cu deposition show negligible changes, indicating the stable structure of surface‐bound CA during catalyst integration. Since the deposition of Cu is driven by photogenerated electrons at CA, Cu disperses at the outside surface of CA without accumulation at the covalent scaffold. In Figure S10 (Supporting Information), the distribution of Cu is thus different from that of other elements (Zr from CA and S from PEDOT/PSS) integrated by thermochemical procedures. In accordance with the SEM images in Figure S10 (Supporting Information), neither CA nor the Cu catalyst block pores of the covalent networks of HrHE‐CA‐Cu.

### 2.2 Photoelectrocatalytic NO_3_^−^ Reduction to NH_3_

**Figure 3:**
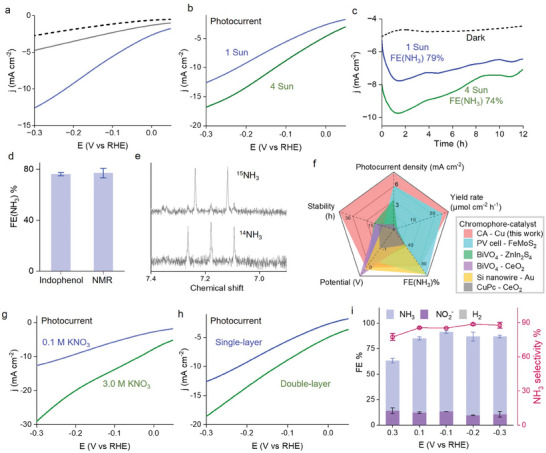
Photoelectrocatalytic NO_3_^−^ reduction to NH_3_. a) Photocurrent densities (j) generated by solar irradiation (100 mW cm^−2^, 1 Sun) of HrHE‐CA‐Cu before (grey line) and after (blue line) charge photoaccumulation. Dashed line: dark current. Electrolyte: argon‐degassed KNO_3_ (0.10 m) in pH 4.5 acetate buffer (1.0 m). b,c) PEC NO_3_^−^ reduction under different solar irradiation intensities. d) Faradaic efficiency of NH_3_ (FE(NH_3_)) estimated using indophenol and ^1^H‐NMR methods. e) Sample ^1^H‐NMR spectra showing the signals of ^15^NH_3_ (upper) and ^14^NH_3_ (lower) produced by feeding the system with ^15^NO_3_^−^ and ^14^NO_3_^−^, respectively. f) A radar plot comparison of different PEC NO_3_^−^ reduction systems between our system and literature‐reported photosystems.^[^ ^25^, ^26^, ^27^, ^28^, ^29^ ^]^ g,h) Photocurrent responses of HrHE‐CA‐Cu showing the influences of NO_3_^−^ concentration (g) and photoelectrode layer (h). i) NH_3_ production efficiencies under various biases.

For the CA photoelectrode, catalytic performances are dictated by efficiencies of both light‐induced charge‐transfer and dark catalytic steps. Solar irradiation of the photocathode generates charge‐separated states with electrons at the conduction band of CA for reducing Cu catalyst, and holes at the valence band for transporting to HrHE and subsequently to the external circuit. The CA photoelectrode generates high cathodic photocurrent densities under solar irradiation at 100 mW cm^−2^ for NO_3_^−^ reduction to NH_3_, as shown in **Figure** **3**a–c. In Figure 3a, pre‐activation of the photoelectrode under irradiation prior to photoelectrocatalysis enhances photocurrent responses due to charge photoaccumulation to facilitate the forward photoinduced charge transfer in Figure 2c. Moreover, the PEC performances are influenced by solar irradiation intensity, NO_3_^−^ concentration and the number of photoelectrode layers (Figure 3; Figures S13 and S14, Supporting Information). In 12‐h photoelectrocatalysis (Figure 3c), the photocathode produces NH_3_ from NO_3_^−^ with cathodic photocurrent density at ≈3.0 mA cm^−2^ and a Faradaic efficiency of NH_3_ (FE(NH_3_)) at 80%. The produced NH_3_ is quantified using both indophenol‐blue method and ^1^H‐NMR spectroscopy coupled with ^15^N‐isotope labeling technique in Figure 3d,e and Figure S15 (Supporting Information). A summary of the PEC efficiencies is given in Table S4 (Supporting Information) based on the reaction steps involving photogeneration of electrons and NO_3_^−^ reduction to NH_3_ by photogenerated electrons. The overall solar‐to‐NH_3_ efficiency (EQE) reaches up to 14%, based on the incident photon flux in Figure S16 (Supporting Information). From the results in Table S4 (Supporting Information), the EQE is dependent on applied bias, which originates from the highly bias‐dependent charge‐separation step to photogenerate reducing equivalents (solar‐to‐electron efficiency: 0.050%–19%). By contrast, the external bias does not significantly influence the electron transfer from the reductively activated catalyst to adsorbed NO_3_^−^, keeping the conversion efficiency in the range of 49%–78%. The total distribution of Faradaic efficiency is less than unity due to generation of non‐catalytic photocurrents during oxidation of reducing intermediates by dissolved O_2_ and trace impurities. Based on the Fermi level of the p‐type electrode substrate (0.86 V vs RHE), the forward photoinduced electron transfer should be thermodynamically feasible under the applied bias range. The bias‐dependent solar‐to‐electron efficiency should result from kinetic reasons since the negatively applied bias helps to deplete the injected holes at PEDOT‐CA interface and hinders charge recombination. The PEC performances in terms of photocurrent, FE(NH_3_), stability and NH_3_ yield rate are compared in the radar plot in Figure 3f (details in Table S5, Supporting Information) between the CA photoelectrode and previously reported PEC NO_3_^−^ reduction systems,^[^ ^25^, ^26^, ^27^, ^28^, ^29^ ^]^ from which, the present charge photo‐accumulating photoelectrode exhibit exceptional performances for NH_3_ photoproduction.

Long‐term photoelectrocatalysis under control conditions including irradiation intensity, NO_3_^−^ concentration and number of photocathode layers, are summarized in Table S6 (Supporting Information). In Figure 3b,c, higher photocurrent density is obtained when the irradiation intensity is increased from 1 Sun (100 mW cm^−2^) to 4 Sun (400 mW cm^−2^), but the external quantum efficiency for NH_3_ production drops to 3.8% (Table S6, Supporting Information) due to the decreased fraction of light absorption by the photoelectrode. Despite increase of photocurrent density with higher NO_3_^−^ concentration in Figure 3g, both FE(NH_3_) and selectivity for NH_3_ production decreases due to production of NO_2_^−^ at FE(NO_2_^−^) of 16% in Figure S13 (Supporting Information), which should result from slow protonation of catalytic intermediates given the relatively low proton concentration at pH 4.5. Correspondingly, photoelectrocatalysis with NO_3_^−^ at low concentration (10 mm) leads to a substantial drop of FE(NH_3_) to 37% (Figure S13, Supporting Information), with serious H_2_ evolution reaction at FE(H_2_) of 42% taking up the photogenerated electrons.

In addition, increasing the number of photoelectrode layers enhances the photocurrent responses (Figure 3h; Figure S14, Supporting Information), however, FE(NH_3_) decreases slightly due to saturation of photon absorption by the millimeter‐thick photoelectrode. It is worth noting that external ionic strengths involving concentrations of the proton buffer and supporting electrolyte influence the PEC performances to some extent (Table S7, Supporting Information). Under small ionic strength with low concentrations of the acetate buffer and supporting electrolyte, the performances are limited by restricted interfacial charge transfer due to insufficient mass transport. The ionic condition is optimized to be a mixture of acetate buffer at 1.0 m with the supporting electrolyte Na_2_SO_4_ at 0.50 m. Owing to favorable electron transfer from the reductively activated catalyst to adsorbed NO_3_^−^, the FE(NH_3_) is not dependent on the applied bias in a relatively wide range in Figure 3i.

The influence of charge accumulation on photoinduced charge‐transfer dynamics are analyzed with results from nanosecond transient absorption (TA) spectra in Figures S17 and S18 (Supporting Information) and kinetics data in Table S8 (Supporting Information). In the absence of the Cu catalyst, photoexcitation of CA creates the excited state, CA^*^, with a lifetime of 2.2 µs which is similar to that of the single chromophore, C (2‐aminoterephthalic acid), 2.4 µs, from fitting of the decay of ground‐state bleach in Figure S17c,d (Supporting Information). By loading the Cu catalyst at CA site, the photogenerated electrons at CA^*^ can be transferred directly to Cu by oxidative quenching at an efficiency of 90% (Figure 2b). The TA trace in Figure S18c (Supporting Information) shows a much longer‐lived charge‐separated state, CA(*h^+^*)‐Cu(*e^−^*), with a lifetime of 28 µs, compared to C(*h^+^*)‐Cu(*e^−^*) with a lifetime of 2.3 µs. The greatly enhanced lifetime of CA(*h^+^*)‐Cu(*e^−^*) results from hole hopping in CA^[^ ^30^, ^31^ ^]^ which acts as a hole reservoir to decelerate charge recombination. In addition, quantum yield of the charge‐separated state, CA(*h^+^*)‐Cu(*e^−^*), is estimated as 87% at 2.0 µs time delay following photoexcitation which is much higher than that of C(*h^+^*)‐Cu(*e^−^*) as 3.3%. The larger density of charge‐separated states provides sufficient reducing equivalents for multielectron reduction of NO_3_^−^.

### 2.3 Influence from Covalent Electrode Substrate

**Figure 4:**
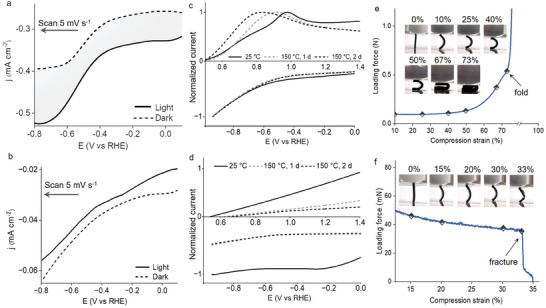
Influence of the covalent networks on the photoelectrochemical behaviors. a,b) Bias‐dependent current densities for HrHE‐CA (a) and HE‐CA (b) under light (solid lines) and dark (dashed lines) in the presence of Cu^2+^ (50 mm). c,d) Electrical conductivity of HrHE (c) and HE (d) as a function of temperatures, illustrating the high stability of HrHE under the CA‐formation temperatures. Upper panel: anodic scan with hydroquinone (30 mm); lower panel: cathodic scan with benzoquinone (30 mm). e,f) Compression process and loading force–strain curve of HrHE (e) and HE (f), illustrating the high flexural and tensile strength of HrHE.

Thermal stability of the covalent electrode substrate is important to allow integration of CA in the networks under elevated synthetic temperatures. For the present photoelectrode structure, the DVB‐crosslinked copolymer of DMA and SS show high thermal stability compared to typical hydrogel systems. The integrated CA photoelectrode exhibits significant cathodic photocurrents in the presence of Cu^2+^ (50 mm) in **Figure** **4**a, demonstrating favorable forward photoinduced electron transfer as illustrated in Figure 2c. By contrast, a control sample, HE, without heat‐resistant components (SS monomer and DVB crosslinker) in the covalent networks does not generate any cathodic photocurrent under the same condition, Figure 4b, which reveals the necessity of using the thermally stable networks for the photocathode. The sharp difference in photoelectrochemical behaviors comes from the hole transporting capability of the electrode substrate. As the electrode substrate of the photocathode, HrHE maintains high hole transporting ability after in situ integration of CA at high temperature, which is evinced by the consistent electrochemical responses of HrHE after heating at 150 °C for 2 days in Figure 4c. By contrast, the control sample HE shows decreased hole conductivity (Figure 4d) under high temperature. In Figures S19 and S20 (Supporting Information), same trends of changes in hole conductivity are observed for HrHE and HE under high temperatures in different solvents including dimethyl sulfoxide (DMSO), dimethylformamide (DMF) and aqueous solutions.

For the thermally stable electrode networks, the high synthetic temperature during CA integration does not change the morphology, as shown by the SEM results in Figure S21a (Supporting Information). By contrast, HE exhibits obvious structural degradation in Figure S21b (Supporting Information). The covalent networks for the CA photoelectrode are demonstrated to withstand temperatures up to 150 °C for a minimum of 48 h with no loss of molecular integrity. Raman spectra in Figure S22 (Supporting Information) reveal negligible structural deviations of HrHE at high temperatures. For HrHE, the interpenetrating networks between PEDOT and the covalent scaffold can confine conformational changes under heating conditions due to strong intermolecular interactions between styrenesulfonate and thiophene moieties of HrHE. In Figure 4e,f, HrHE exhibit much greater mechanical stability with high tensile strength relative to HE during compression processes, demonstrating the high structural stability of HrHE. Under loading forces, HrHE strains progressively to reach the highest flexural deformation point (73%) without breaking, while HE breaks by compression strain at 33%. Based on those observations, the highly stable hole‐transporting covalent networks provide a basis for in situ integration of the photosensitizer, CA, without loss of PEC performances of the resulting photoelectrode.

### 2.4 Multielectron activation of Cu catalyst

**Figure 5:**
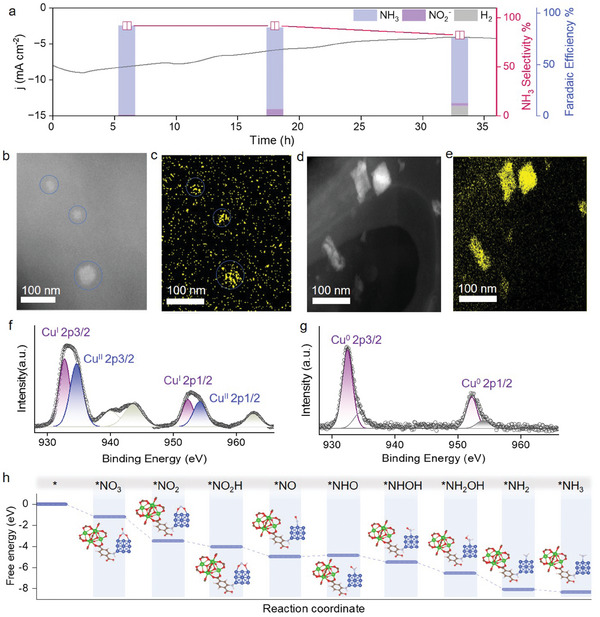
Catalyst photoactivation during long‐term photoelectrocatalysis. a) Continuous PEC NO_3_^−^ reduction to NH_3_ by solar irradiation (100 mW cm^−2^) of HrHE‐CA‐Cu. b–e) High‐angle annular dark‐field scanning transmission electron microscopy (HAADF‐STEM) images for the photocathode before (b,c) and after (d,e) charge photoaccumulation. f,g) XPS spectra for Cu catalyst before (f) and after (g) activation. The XPS spectrum in (g) was obtained after 12‐h solar irradiation (1 Sun) of the photoelectrode at −0.30 V vs RHE in argon‐degassed KNO_3_ (0.10 m) electrolyte at pH 4.5. h) Energy barriers from DFT calculation showing the evolution of intermediates during NO_3_^−^ reduction to NH_3_.

Solar irradiation of the CA photocathode produces NH_3_ from NO_3_^−^ continuously for at least 36 h with a small decrease in FE(NH_3_) and catalytic selectivity in **Figure** **5**a, verifying the robustness of the photoelectrode. During the long‐term photoelectrocatalysis, morphologies of the covalent electrode networks and CA do not exhibit apparent changes, as shown by the SEM and EDX results in Figures S23 and S24 (Supporting Information). During photoelectrocatalysis, the Cu catalyst is activated by photogenerated electrons accumulated at the conduction band of CA with a reducing power of −0.37 V vs NHE. The activation process is analyzed by changes in oxidation states and morphology of Cu, as revealed respectively by high‐angle annular dark‐field scanning transmission electron microscopic (HAADF‐STEM) results in Figure 5b–e and XPS spectra in Figure 5f,g. In the initial state prior to photoelectrochemical activation, the Cu catalyst exhibits spherical morphology with an average diameter of 40–50 nm (Figure 5b). The XPS spectrum in Figure 5f illustrates the presence of Cu at relatively high oxidation states in the initial state, from signals for Cu^II^ (2p_3/2_ at 934.6 eV and 2p_1/2_ at 954.3 eV) and Cu^I^ (2p_3/2_ at 932.7 eV and 2p_1/2_ at 952.4 eV). After photoelectrochemical activation, the Cu catalyst shows morphological changes to sheet particles in Figure 5d,e, which is accompanied with a decrease in its oxidation states shown by the XPS signals of Cu^0^ (2p_3/2_ at 932.5 eV and 2p_1/2_ at 952.2 eV) in Figure 5g. Taking account of the reduction potential of Cu^2+/0^ at 0.34 V vs NHE and the conduction band edge potential of CA at −0.37 V vs NHE, reductive activation of the Cu catalyst is thermodynamically feasible with a large driving force. During the long‐term PEC experiment, a small portion of the Cu catalyst (4.0% of the initial amount) leaks to the electrolyte based on inductively coupled plasma‐optical emission spectroscopic (ICP‐OES) results in Table S9 (Supporting Information). By examination of both the photoelectrode and the electrolyte after photoelectrocatalysis with Raman and ^1^H‐NMR spectroscopies (Figures S25 and S26, Supporting Information), it can be concluded that the covalent network of the photoelectrode is structurally stable under the long‐term PEC conditions. The pore size of HrHE‐CA‐Cu after photoelectrocatalysis varies negligibly (Table S1, Supporting Information), confirming the stable morphology of the entire photoelectrode.

Solar irradiation of CA accumulates electrons at the excited states that go exclusively to NO_3_^−^‐adsorbed Cu catalyst to result in the eight‐electron reduced product, NH_3_. The PEC selectivity for NH_3_ is quite high at 92% (Table S6, Supporting Information). Hydrogen evolution reaction (HER) as one of typical side reactions is suppressed, which is rationalized by the density functional theory (DFT) calculations with results given in Figure 5h and Figure S27 (Supporting Information). A simplified model for the chromophore‐modified Cu catalyst is adopted here based on structural optimization with 13 Cu atoms.^[^ ^32^ ^]^ The energy barriers for elemental steps in NO_3_^−^‐to‐NH_3_ conversion are summarized in Figure 5h. From Figure S27 (Supporting Information) and Figure 5h, adsorption of NO_3_^−^ on the Cu catalyst (−1.40 eV) is much more favorable than adsorption of H^+^ (−0.46 eV), confirming the ineffectiveness in HER by the photoelectrode. The projected density of states in Figure S27 (Supporting Information) show a more positive d‐band center^[^ ^33^ ^]^ for Cu‐^*^NO_3_^−^ (−2.04 eV) than Cu‐^*^H (−2.16 eV), which is caused by the stronger interaction between ^*^NO_3_^−^ and Cu. It is worth noticing that the calculational results for the chromophore‐modified Cu and the pristine Cu are different. As shown in Figure S28 (Supporting Information), the pristine Cu has more positive energies for both ^*^NO_3_^−^ (−0.98 eV) and ^*^H (−0.27 eV) adsorption, compared with those of the chromophore‐modified Cu (Figure S27, Supporting Information). The results, together with the more negative d‐band centers in Figure S28 (Supporting Information), indicate a beneficial effect of the chromophore modification in promoting NO_3_^−^ adsorption on Cu. For the photoelectrode, solar irradiation accumulates electrons at CA excited states that transfer to Cu adsorbed with NO_3_^−^ and other intermediate species successively in Figure 5h, minimizing loss in photogenerated electrons and enhancing the overall PEC efficiency.

## 3 Conclusion

In summary, we introduce a charge accumulating photoelectrode whose photosensitizing component is a chromophore assembly composed of zirconium‐coordinated 2‐aminoterephthalic acid. In contrast to single‐molecule sensitized systems, the photoelectrode generates high‐density electrons located at the assembly for transferring to an adjacent Cu catalyst toward eight‐electron reduction of NO_3_^−^ to NH_3_. For the photoelectrode, integration of the chromophore assembly under elevated temperatures is enabled by constructing thermally stable, hole‐transporting covalent networks as the electrode substrate. The CA photoelectrode shows unique features such as high scalability in three dimensions, durable mechanical properties owing to the covalent scaffold, and high hydrophilicity that is advantageous for aqueous catalytic applications.

The photoelectrode exhibits high PEC performances, with a high external quantum efficiency for solar‐driven NO_3_^−^‐to‐NH_3_ conversion at 14%, stable cathodic photocurrent density at 3.0 mA cm^−2^ for tens of hours, and Faradaic efficiency for NH_3_ production at 80%. Investigation of the electrode substrate reveals that the high hole conductivity of the thermally stable covalent networks provides a basis for favorable forward charge transfer for catalyst activation. This work provides an effective strategy for catalytic photoelectrodes that is mechanistically distinct from previously developed systems. By introducing the new photoelectrode structure, we show that multi‐electron redox catalysts can be effectively activated by charge photo‐accumulating assembly anchored in covalent networks for high‐performance PEC cells. Future structure‐based engineering of the CA photoelectrodes can be involved to further improve solar‐to‐chemical conversion efficiencies toward commercially viable PEC devices.

## Conflict of Interest

The authors declare no conflict of interest.

## Supporting information

Supporting Information

## Data Availability

The data that support the findings of this study are available from the corresponding author upon reasonable request.
