# Supplementary material for: Charge Photoaccumulation in Covalent Polymer Networks for Boosting Photocatalytic Nitrate Reduction to Ammonia
Source: Adv Sci (Weinh). 2024 Apr 6;11(23):2401878. doi: 10.1002/advs.202401878 (PMC11187893; doi:10.1002/advs.202401878)
Supplement: Supplementary file 1 — Supporting Information [file ADVS-11-2401878-s001.pdf]

ADVANCED SCIENCE
Open Access

Supporting Information

for *Adv. Sci.*, DOI 10.1002/advs.202401878

Charge Photoaccumulation in Covalent Polymer Networks for Boosting Photocatalytic Nitrate Reduction to Ammonia

*Xinjia He, Yingke Wen, Yanjie Fang, Mengjie Li and Bing Shan**

Supporting Information

Charge Photoaccumulation in Covalent Polymer Networks for Boosting Photocatalytic Nitrate Reduction to Ammonia

Xinjia He,[1,3] Yingke Wen,[1,3] Yanjie Fang,[1] Mengjie Li,[1] Bing Shan,[1,2,*]

[1] Department of Chemistry, Zhejiang University, Hangzhou 310058, China

[2] Key Laboratory of Excited-State Materials of Zhejiang Province, Hangzhou 310058, China

[3] These authors contributed equally

* E-mail: bingshan@zju.edu.cn

Contents

Experimental section--3-7
Figure S1. Synthetic procedure for HrHE -- 8
Figure S2. FTIR spectra for DMA, NaSS and HrH--- 9
Figure S3. Hole conductivity controlled by different polymerization conditions ----------------10
Figure S4. Synthetic procedure for HrHE-CA-Cu ---11
Figure S5. XRD patterns for CA---12
Figure S6. XPS spectra for CA---13
Figure S7. Chemical structure and physical properties--14
Figure S8. Normalized absorption spectra of C and CA --15
Figure S9. Band potential evaluations for PEDOT in HrHE ---------------------------------------16
Figure S10. SEM with EDX for HrHE-CA and HrHE-CA-Cu ---------------------------------------17
Figure S11. XPS spectra for HrHE-CA ---18
Figure S12. XPS spectra for HrHE-CA-Cu--19
Figure S13. NO_3^- concentration-dependent PEC performances -----------------------------------20
Figure S14. 12-hour PEC with single- and double-layer samples----------------------------------21
Figure S15. ^{15}N isotope labelling experiment --22
Figure S16. Simulated solar irradiation spectrum ---23
Figure S17. TA spectra for CA and C --24
Figure S18. TA spectra for CA-Cu and C-Cu --25
Figure S19. Electrochemistry for HrHE at 100 °C in different solvents ----------------------------26
Figure S20. Electrochemistry for HE at 100 °C in different solvents -------------------------------27
Figure S21. SEM for HrH and HS under high temperatures --28
Figure S22. Raman spectra for HrHE before and after heating--------------------------------------29
Figure S23. Post-PEC SEM and EDX spectra for the photoelectrode -------------------------------30
Figure S24. EDX spectra for the photoelectrode before and after PEC------------------------------31
Figure S25. Raman spectra for HrHE-CA-Cu before and after PEC ---------------------------------32
Figure S26. ^{1}H-NMR spectra for electrolytes before and after PEC---------------------------------33
Figure S27. PDOS for nitrate- and proton- adsorbed catalyst--34
Figure S28. PDOS for nitrate- and proton- adsorbed on Cu catalyst ----------------------------------35
Table S1. Summaries of contact angles, pore sizes and porosities ----------------------------------36
Table S2. Band edge potentials of CA and HrHE --37
Table S3. Surface coverages --38
Table S4. Photoelectrocatalytic efficiencies for the CA photocathode ------------------------------39
Table S5. Comparisons on solar-to-NH_3 performances--40
Table S6. Photoelectrocatalytic performances ---41
Table S7. Ionic-limiting factors that influence the PEC efficiencies --------------------------------42
Table S8. TA Fitting parameters --43
Table S9. The percentage of metal leakage during long-term PEC ----------------------------------44
References---45

Experimental Section

Synthesis of the electrode substrates

The synthetic procedure for HrHE is illustrated in Figure S1. The HrH scaffold was synthesized by thermal-initiated radical copolymerization [1] of DMA (3.29 M) and sodium 4-vinylbenzenesulfonate (0.17 M) in a mixed solvent of DMSO and deionized H_2O (volume ratio: 1:1), with DVB (0.10 M) as the crosslinker and 2,2'-azobis[2-(2-imidazolin-2-yl)propane] dihydrochloride (35 mM) as the thermal initiator. The solution was transferred to a custom-made silicon template and sealed with glass. The solution was subject to heating at 35 °C for about two hours to obtain HrH. After removing from the template, HrH was thoroughly washed with deionized H_2O, and subsequently soaked in a methanol solution of 3,4-ethylenedioxythiophene (EDOT) (0.50 M) for 2 hours to pre-saturate the film with EDOT. PEDOT was integrated in HrH by in situ polymerization of EDOT for 24 hours with iron (III) p-toluenesulfonate (1.0 M) as the oxidative initiator. HrHE was obtained after rinsing with methanol to remove unreacted reagents and byproducts. The covalent scaffold without heat-resistant components was synthesized by photoinitiated radical polymerization of DMA (3.0 M) in deionized H_2O with N,N′-methylenebis(acrylamide) (30 mM) as a chemical crosslinker and a photoinitiator, 2-hydroxy-4'-(2-hydroxyethoxy)-2-methylpropiophenone (30 mM) under UV light (365 nm) irradiation for an hour. The procedure for PEDOT integration to generate HE is the same as that for HrHE.

Synthesis of the photocathode HrHE-CA

In-situ integration of monodispersing CA (CA) particles generates the photocathode, HrHE-CA, as illustrated in Figure S4. Before CA synthesis, the HrHE film was pre-saturated with a DMF solution of 2-aminoterephthalic acid (H_2ATA) (75 mM) and $ZrCl_4$ (50 mM) with an acidic modulator, HCl (5% v/v), for 6 hours, followed by in-situ formation of CA in HrHE networks under 120 °C for 6 hours. The photocathode, HrHE-CA, was obtained after rinsing with excess DMF and drying under vacuum for 12 hours.

Catalytic functionalization of the photocathode

The photocathode was functionalized with the Cu catalyst based on a modified photochemical approach reported previously.[14] Briefly, the CA photocathode, HrHE-CA, was pre-saturated with the deposition solution containing $CuSO_4$ (50 mM, aq.) for 6 hours, followed by photoelectrochemical deposition in the same solution for 7200 s under an applied bias at -0.3 V vs RHE. The CA photocathode, a platinum mesh and a Ag/AgCl electrode were used as the working, counter and reference electrodes, respectively, in an H-type electrolysis cell with a proton-exchange membrane (Nafion N117). Light illumination was provided by an AM 1.5, 1 Sun solar simulator (Beijing China Education Au-light Co. CEL-S500 with an AM 1.5 filter) whose intensity was calibrated using a standard Si cell. The deposition solution was degassed with Ar for at least 30 minutes prior to experimentation. After photoelectrochemical deposition, the film was rinsed with argon-degassed, deionized H_2O to remove unreacted ions.

Characterizations for the covalent networks

The microstructures of the electrodes were analyzed by scanning electron microscopy (SEM, Hitachi SU8010), transmission electron microscopy (TEM, HT7700) and high-angle annular dark-field scanning transmission electron microscopy (HAADF-STEM, JEM 2100F) with energy dispersive X-ray spectrometry (EDX). The electrodes were freeze-dried prior to those measurements. Contact angles were measured at room temperature with a DataPhysics OCA-15EC analyzer. Compression tests were performed by using a tensile tester (Instron, 3343) equipped with a 50 N-force sensor with loading velocity at 2.0 mm/min in air.

Photoelectrocatalytic experiments

The PEC experiments were performed using a typical three-electrode, H-type electrolysis cell with a proton-exchange membrane (Nafion, N117) separating cathodic and anodic compartments. In the reaction cell, the cathodic compartment contains the CA photocathode as the working electrode that is irradiated by the solar simulator (Beijing China Education Au-light Co. CEL-S500 with an AM 1.5 G filter) with light intensity calibrated by a standard Si cell. The anodic compartment was equipped with a platinum mesh and a Ag/AgCl electrode as the counter and reference electrodes, respectively. The electrolyte solution for a typical PEC experiment contains KNO_3 (0.10 M) in argon-degassed acetate buffer (1.0 M, pH 4.5) with Na_2SO_4 (0.50 M) added as a supporting electrolyte. Potentiostats (CH Instruments, 760E, 660E or 920D) were used to collect photocurrent responses and provide biases to the cell. The recorded potential was converted to reversible hydrogen electrode (RHE) scale as follows: E (vs RHE) = E (vs Ag/AgCl) + 0.197 V + 0.059 pH. After the PEC experiments, the gas products were analyzed by a gas chromatography system (Shimadzu Nexis GC-2030) equipped with both thermal-conductivity and flame-ionization detectors. The liquid products were analyzed by ^{1}H-NMR spectroscopy and spectrophotometric methods (*vide infra*).

Photoelectrocatalytic efficiency

The faradaic efficiency (FE) of H_2, NO_2^- and NH_3 was calculated as follows:

$$\mathrm{FE\ (H_2)} = \frac{2\times \mathrm{n(H_2)}\times \mathrm{F}}{\mathrm{Q}}$$

$$\mathrm{FE\ (NO_2^-)} = \frac{2\times \mathrm{n(NO_2^-)}\times \mathrm{F}}{\mathrm{Q}}$$

$$\mathrm{FE\ (NH_3)} = \frac{8\times \mathrm{n(NH_3)}\times \mathrm{F}}{\mathrm{Q}}$$

The yield rate of NH_3 was calculated as follows:

$$\mathrm{Yield\ rate\ (NH_3)} = \frac{\mathrm{n(NH_3)}}{\mathrm{t}}$$

The selectivity of NH_3 was calculated as follows:

$$\mathrm{Selectivity\ (NH_3)} = \frac{\mathrm{FE\ (NH_3)}}{\mathrm{FE\ (NH_3)+FE\ (H_2)+FE\ (NO_2^-)}}$$

The external quantum efficiency (EQE) was calculated as follows:

$$\text{EQE} = 8\times\frac{\text{Moles of NH}_3}{\text{Moles of incident photons}}$$

where F is the Faraday constant, n (x) (x: H_2, NO_2^-, NH_3) is the moles of the product, Q is the total charge passing the electrode, and t is the photoelectrocatalysis time. Background products and currents were subtracted using the same experimental setup in the absence of the samples.

Quantification of ammonia (NH_3)

The produced NH_3 was quantified by using both indophenol-blue method [2] and ^{1}H-NMR spectroscopy. For the indophenol-blue method, an aliquot of the electrolyte was taken out from the cathodic compartment and diluted to the detectable range. Typically, 1.0 mL of the diluted electrolyte was mixed with a solution (1.0 mL) containing sodium hydroxide (1.0 M), salicylic acid (5.0 wt%) and sodium citrate (5.0 wt%). The resulting solution was added with sodium hypochlorite (0.50 mL, 0.05 M) and sodium nitroferricyanide (0.10 mL, 1.0 wt%). After sitting in the dark for 2 hours, the solution mixture was examined by UV-Vis absorption spectroscopy using a spectrophotometer (Shimadzu UV-2600i). The concentration of the produced NH_3 was determined based on the absorbance at 650 nm using the calibration curve in the following figure. For accurate assessment, the produced NH_3 was also quantitatively determined by ^{1}H-NMR spectroscopy (Agilent DD2 600 MHz) using DMSO-d_6 as the solvent and maleic acid ($C_4H_4O_4$) as an internal standard.[2-4] Typically, the diluted electrolyte (125 μL) was mixed with maleic acid (125 μL, 100 μM in DMSO-d_6), sulfuric acid (50 μL, 4.0 M in DMSO-d_6) and DMSO-d_6 (750 μL). The NH_3 concentration was obtained from the characteristic peak area (relative to the internal standard) according to the calibration curve given in the following figure. In order to investigate nitrogen source for the produced NH_3, ^{15}N isotope-labeling experiment was conducted by replacing $K^{14}NO_3$ with $K^{15}NO_3$ (98 atom% ^{15}N) in the electrolyte for the PEC experiments. The commercial $K^{15}NO_3$ was pre-purified to remove possible impurities of $^{14}NH_4^+$ or $^{15}NH_4^+$. The cathodic electrolyte was examined by ^{1}H-NMR spectroscopy as detailed above to estimate the amount of $^{15}NH_4^+$. The ^{1}H-NMR specta for $^{15}NH_4^+$ show doublet at 7.12 and 7.24 ppm, and for $^{14}NH_4^+$ show triplet at 7.09, 7.18 and 7.26 ppm. The signal for the internal standard, maleic acid, appears at 6.25 ppm. Calibration data for the indophenol-blue and ^{1}H-NMR methods are shown below for $^{14}NH_4^+$ (a, b) and $^{15}NH_4^+$ (c, d).

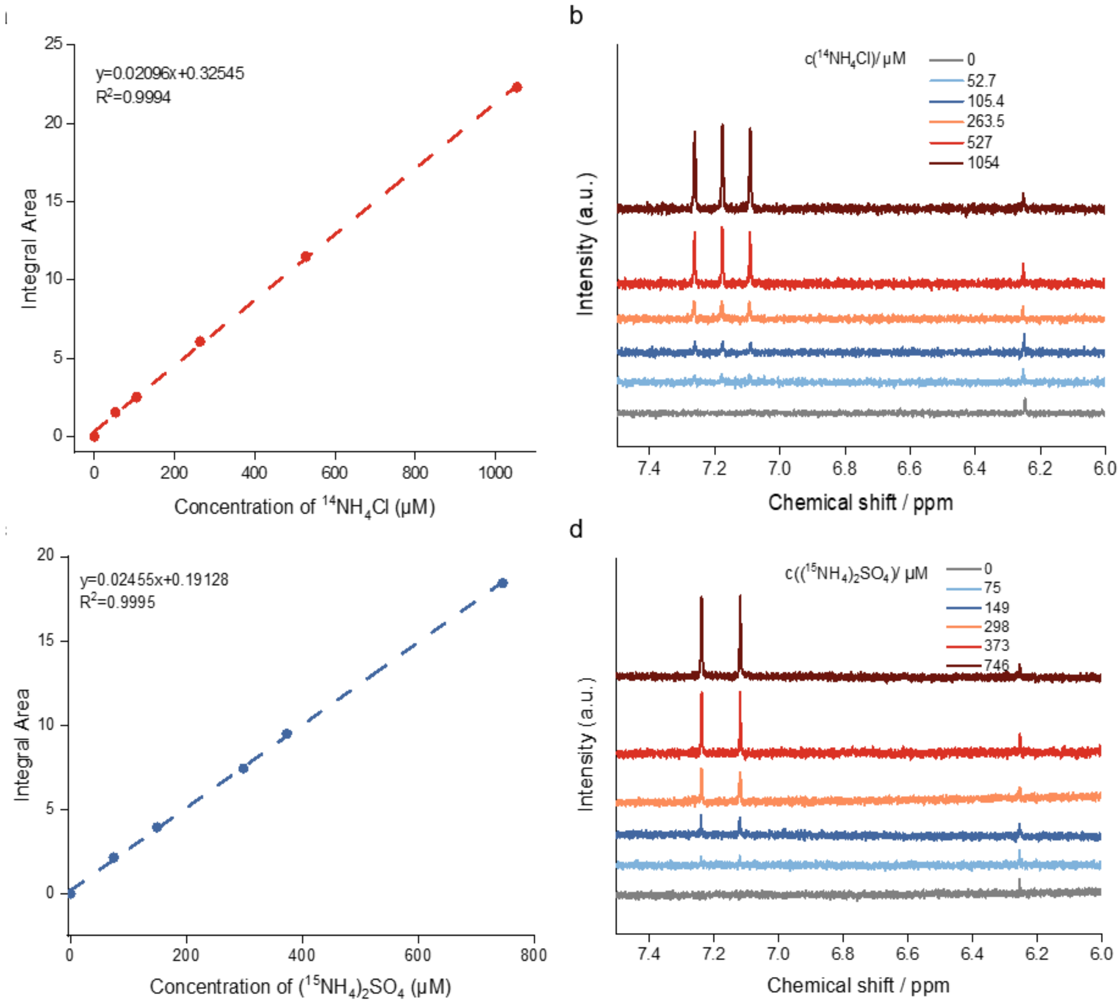

Quantification of nitrite (NO_2^-)

The amount of produced NO_2^- was determined by Griess test.[5] Two reagent solutions (denoted as A and B) were prepared before the test. For solution A, *p*-aminobenzene sulfonamide (1.0 g) was dissolved in H_3PO_4 aqueous solution (100 mL, 10 vol.%). For solution B, N-(1-Naphthyl) ethylenediamine dihydrochloride (0.10 g) was dissolved in H_2O (100 mL). An aliquot of the diluted electrolyte (1.0 mL) was mixed with solutions A and B of 0.50 mL each. After sitting in the dark for 20 minutes, the solution mixture was examined by UV-Vis absorption spectroscopy using the spectrophotometer (Shimadzu UV-2600i). The following figure shows the calibration data by spectroscopic measurements for NH_4^+ (a,b) and NO_2^- (c,d). The concentration of NO_2^- was determined based on the absorbance at 540 nm using the calibration curve in the following figure (c,d).

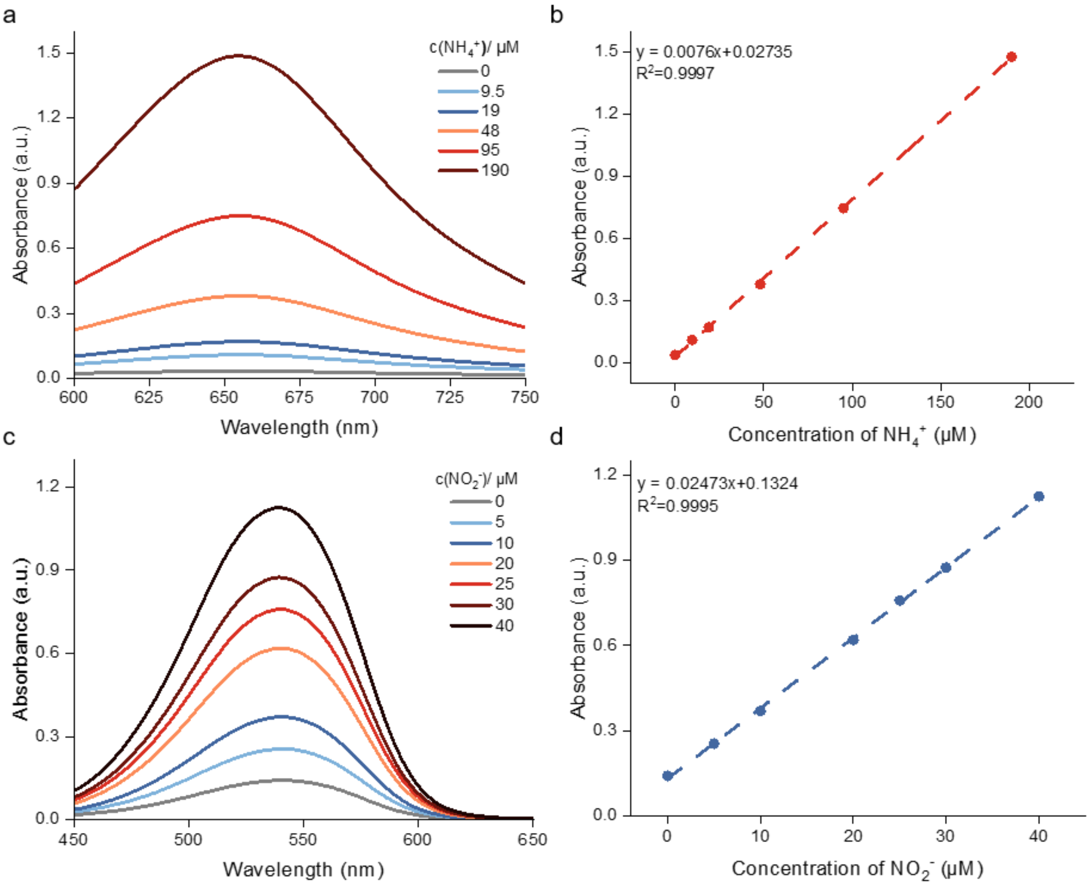

DFT calculations

VASP code was adopted for DFT calculations for the catalytic processes.[6] In this method, Perdew–Burke–Ernzerhof functional within generalized gradient approximation[7] was used to process the exchange–correlation. The project augmented-wave pseudopotential[8] was applied with a kinetic energy cut-off at 500 eV, which was utilized to describe the expansion of the electronic eigenfunctions. The vacuum thickness was set to be 15 Å to minimize interlayer interactions.[9] The Brillouin-zone integration was sampled by a Γ-centered 2 × 2 × 1 Monkhorst–Pack k-point. All atomic positions were fully relaxed until the energy and force reached a tolerance of 1.0×10^{-5} eV and 0.030 eV/Å, respectively. The dispersion corrected DFT-D method was employed to consider the long-range interactions. The D-band center was determined using the equation, $\varepsilon_d = \frac{\int_{-\infty}^{\infty} n_d(\varepsilon)\varepsilon d\varepsilon}{\int_{-\infty}^{\infty} n_d(\varepsilon) d\varepsilon}$, where ε and $n_d(\varepsilon)$ represent the energy level and DOS for the d orbitals, respectively.

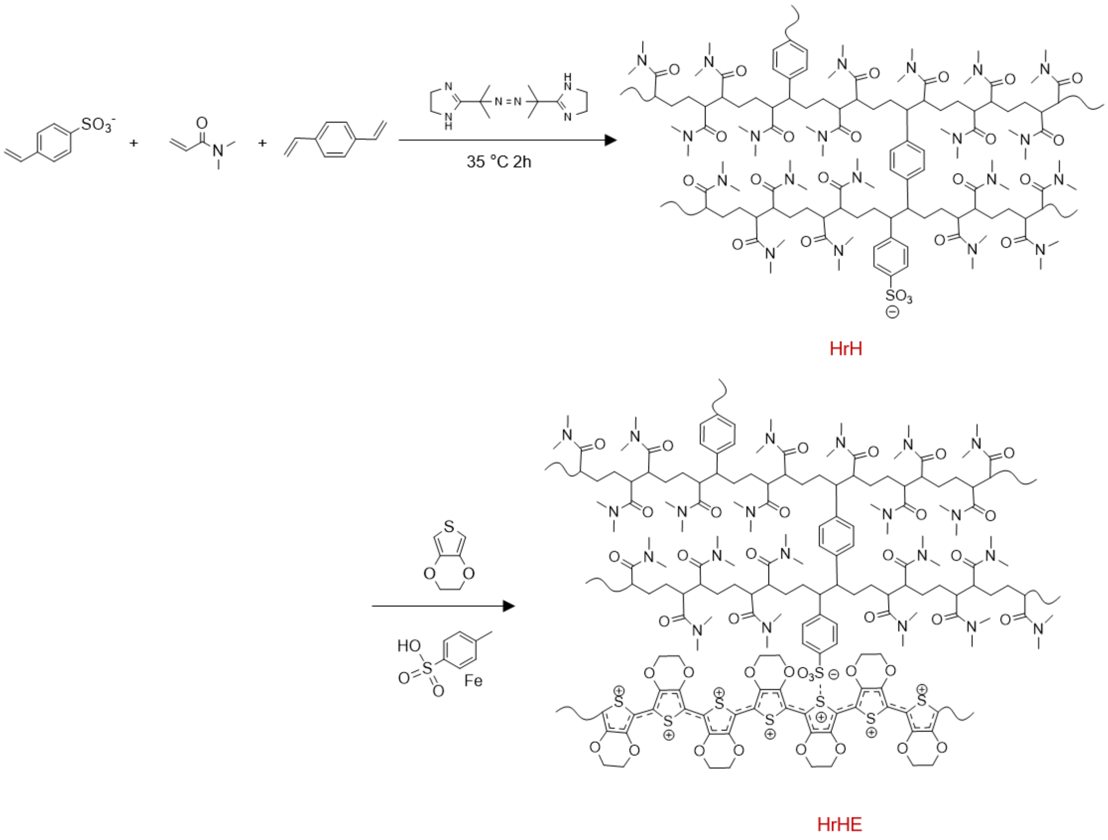

Figure S1. Synthetic procedure for the covalent electrode substrate (HrHE).

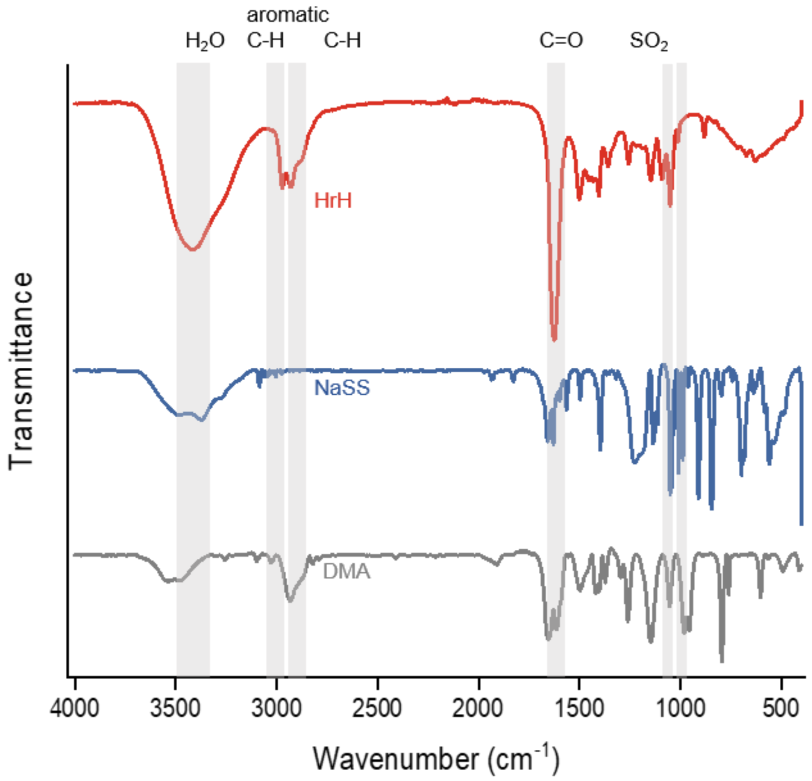

Figure S2. Fourier-transform infrared (FTIR) spectra for DMA, NaSS monomer and HrH, showing the successful combination of NaSS and DMA in HrH.

The FTIR spectrum for HrH (red curve in Figure S2) displays stretching vibrations of C–H at 2928 cm^{-1} and O-H of H_2O adsorbed in the covalent networks at 3450 cm^{-1}.[10-11] The disappearance of the C=C peak at 1610 cm^{-1}, compared with the DMA monomer (gray curve), indicates polymerization of DMA to form HrH. In addition, the amide peak at 1648 cm^{-1} shifts to 1616 cm^{-1} due to formation of hydrogen bonds in HrH. The characteristic peaks at 1010 cm^{-1}, 1040 cm^{-1} (symmetric SO_2 stretching) and 2974 cm^{-1} (aromatic C-H stretching) confirm copolymerization of DMA and NaSS.

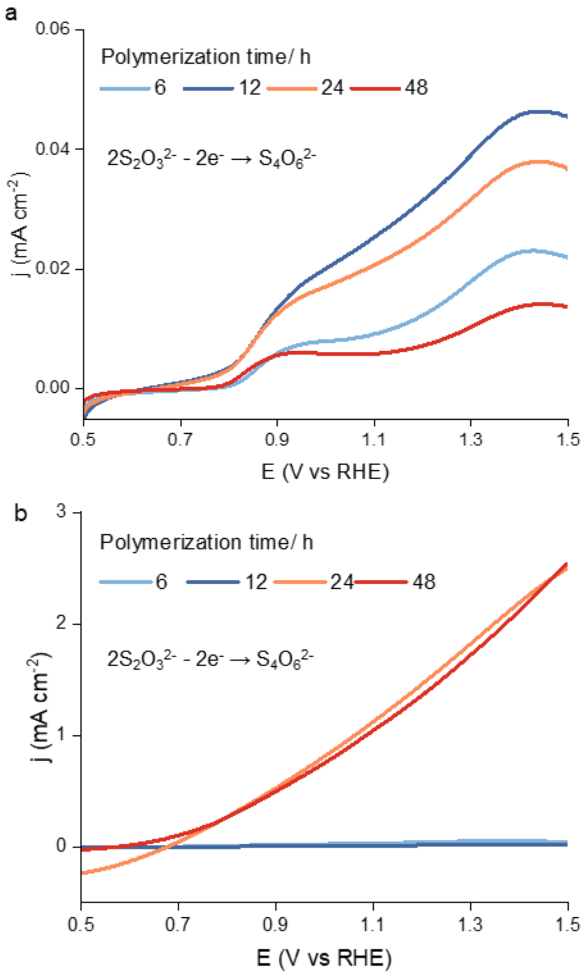

Figure S3. Linear sweep voltammograms for HrHE in pH 4.5 acetate buffer (0.10 M) with $Na_2S_2O_3$ (20 mM). Scan rate: 5.0 mV s^{-1}. The Faradaic currents come from oxidation of $S_2O_3^{2-}$ to $S_4O_6^{2-}$.[12] The HrHE samples were prepared by polymerizing EDOT at 0.30 M (**a**) and 0.50 M (**b**) as a function of polymerization time.

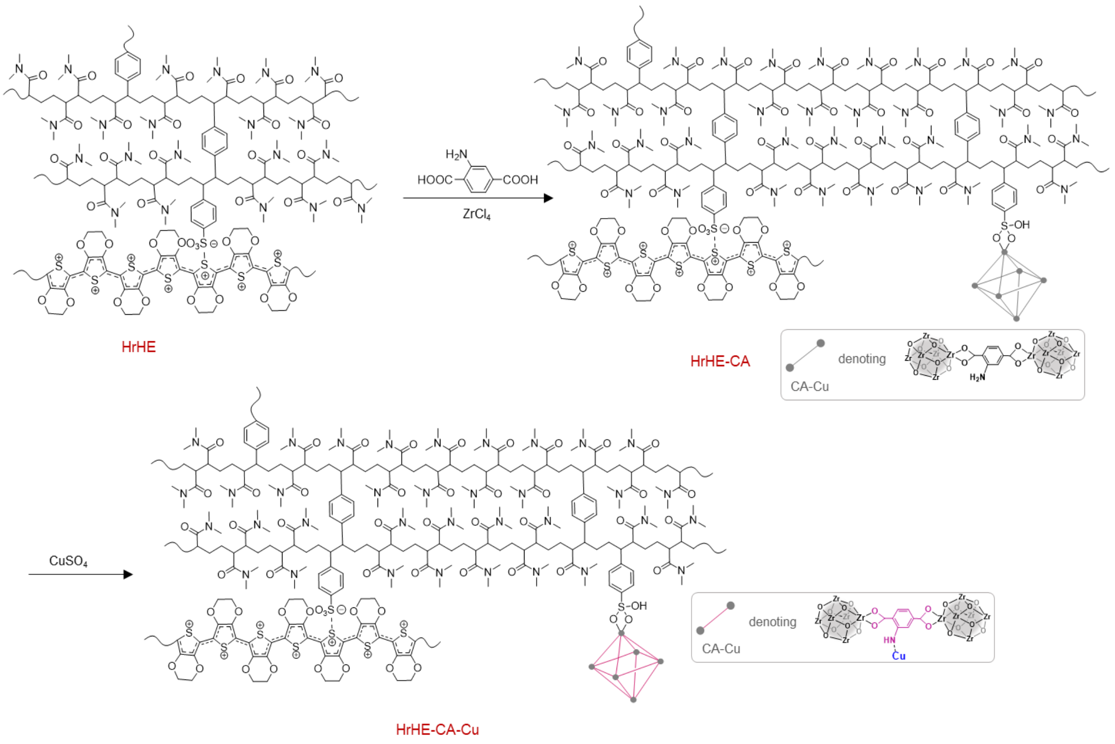

Figure S4. Synthetic procedure for the photocathode with Cu solid catalyst (HrHE-CA-Cu). The Cu catalyst was loaded by site-selective photoelectrochemical deposition at CA in HrHE-CA photocathode (see Methods for the synthetic details).

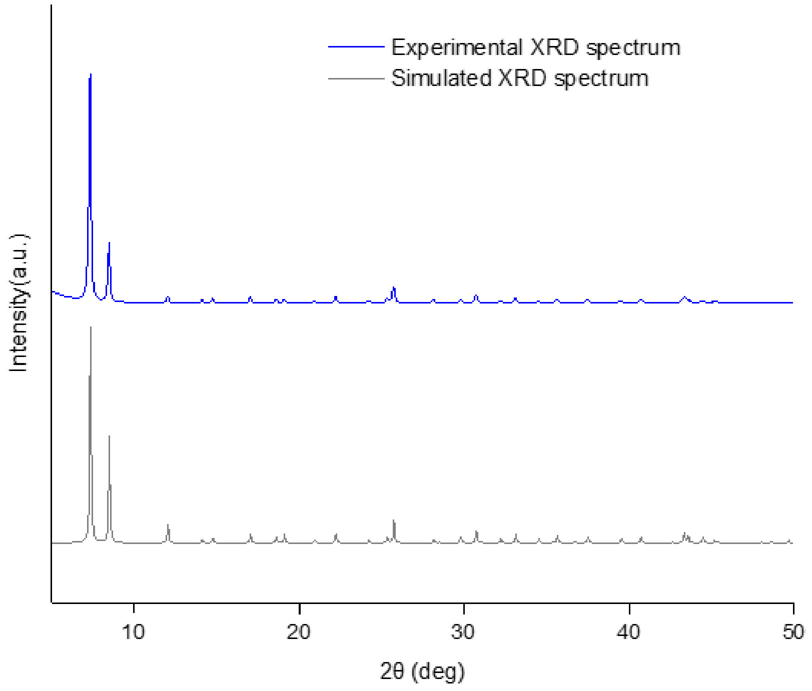

Figure S5. X-ray diffraction patterns (XRD) for CA in the CA photocathode.

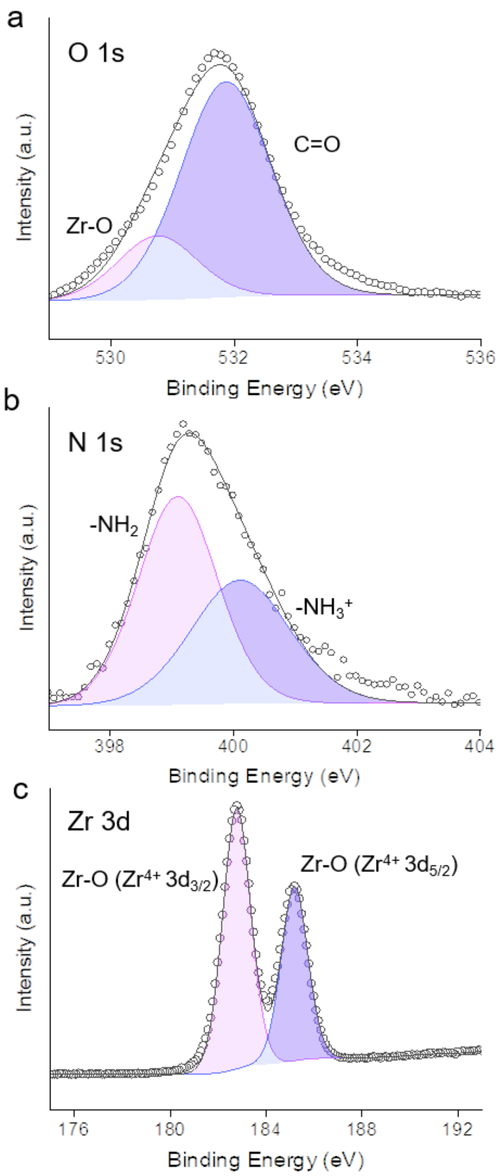

Figure S6. X-ray photoelectron spectroscopy (XPS) spectra for CA in the CA photocathode. a, O 1s; **b**, N 1s; **c**, Zr 3d.

The high-resolution spectrum of O 1s (**a**) displays the contributions of the O species associated with the linker units. The deconvolution revealed the prominent presence of C=O (531.9 eV) and Zr-O (530.8 eV) species. The spectrum of N 1s (**b**) shows the contribution of $–NH_2$ bonded to the phenylene of the linker ($–NH_2$, 399.1 eV) and the protonated amidogen form ($–NH_3^+$, 400.1 eV). The two peaks in the spectrum of Zr 3d (**c**) at 182.8 and 185.1 eV are attributed to Zr^{4+} $3d_{5/2}$ and $3d_{3/2}$, respectively, suggesting the formation of Zr-O bonds in the metal cluster.[13]

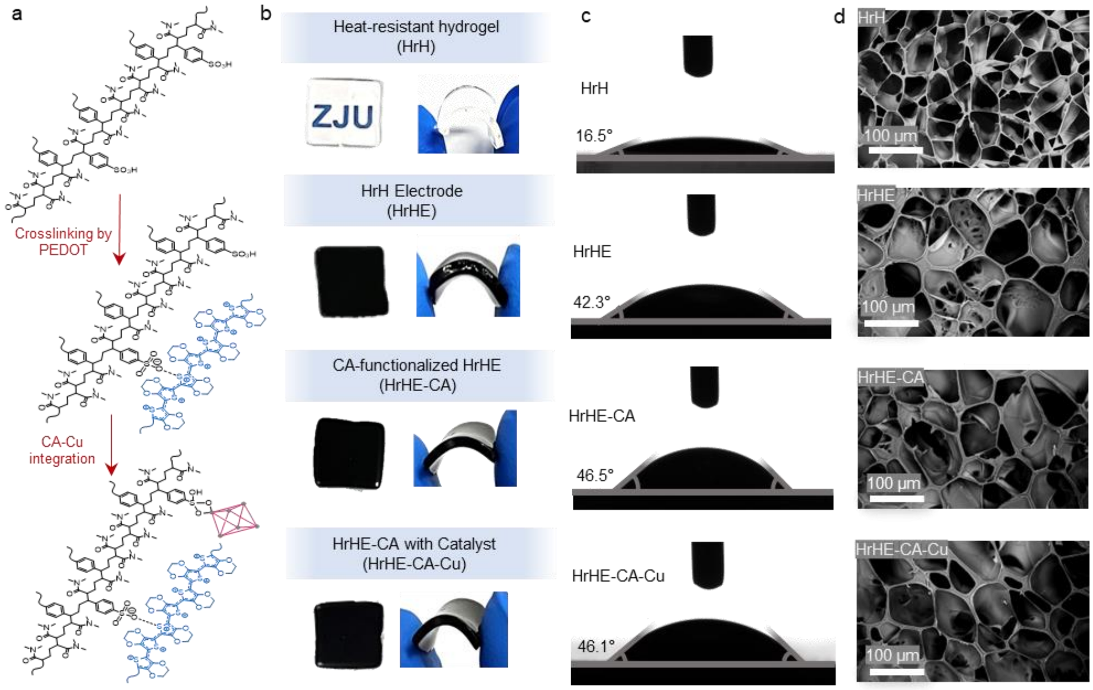

Figure S7. Molecular photocathode constructed with heat-resistant conductive networks. a, Structure of the CA photocathode (HrHE-CA-Cu). **b-d,** Optical images (**b**), contact angles (**c**) (water drop volume: 3 μL) and scanning electron microscopy (SEM) micrographs (**d**) for the heat-resistant hydrogel scaffold (HrH), the hydrogel electrode substrate (HrHE), the photocathode (HrHE-CA) and the photocathode with Cu catalyst (HrHE-CA-Cu).

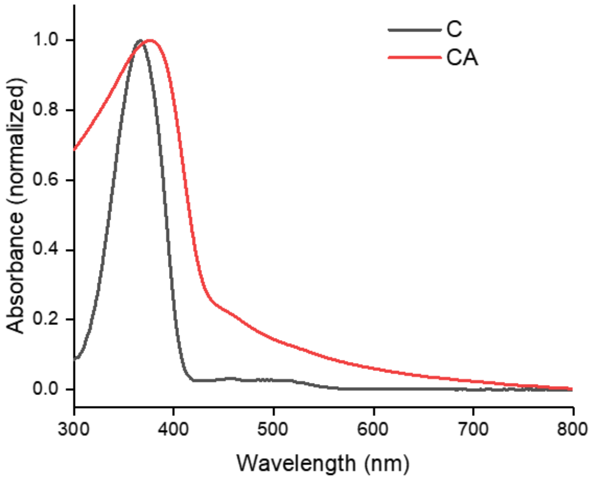

Figure S8. Normalized absorption spectra of C and CA. Samples were dispersed in DMF for measurement.

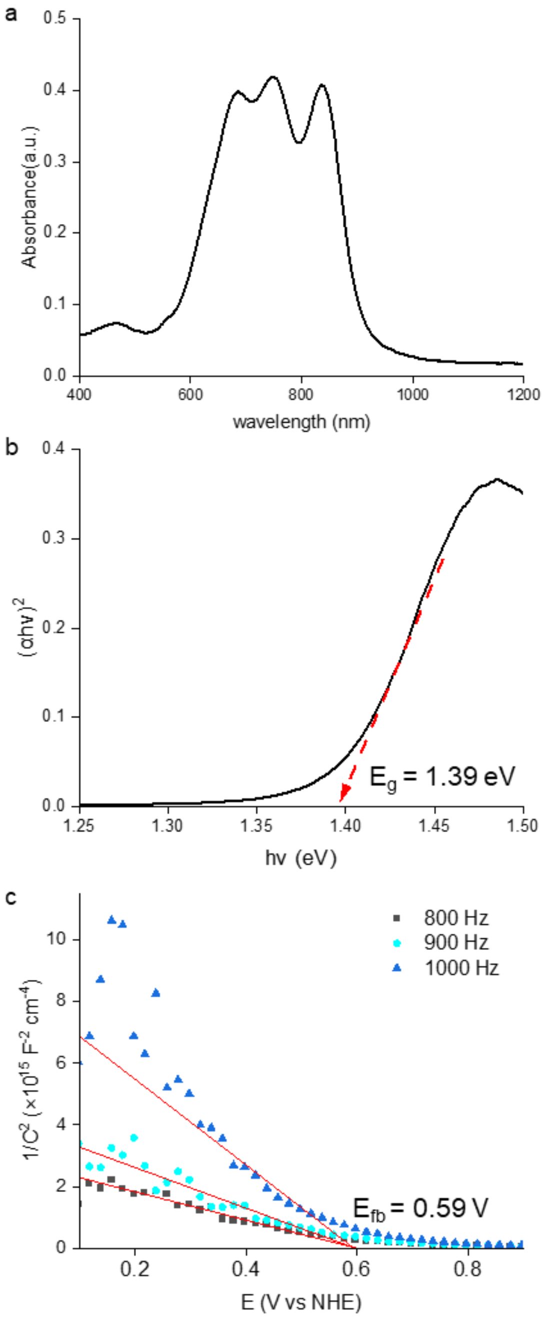

Figure S9. UV-vis absorption spectra (a), Tauc plot (b) and Mott−Schottky plots (c) for PEDOT. The band gap and flat band potential of PEDOT are determined to be 1.39 eV and 0.59 V, respectively. For the Mott–Schottky measurements, the voltage range was 0.90 to 0.10 V (vs NHE) with an amplitude of 0.005 V at frequencies of 800, 900, 1000 Hz. The recorded potential was converted to normal hydrogen electrode (NHE) according to $E_{NHE} = E_{Ag/AgCl} + 0.197$ V.

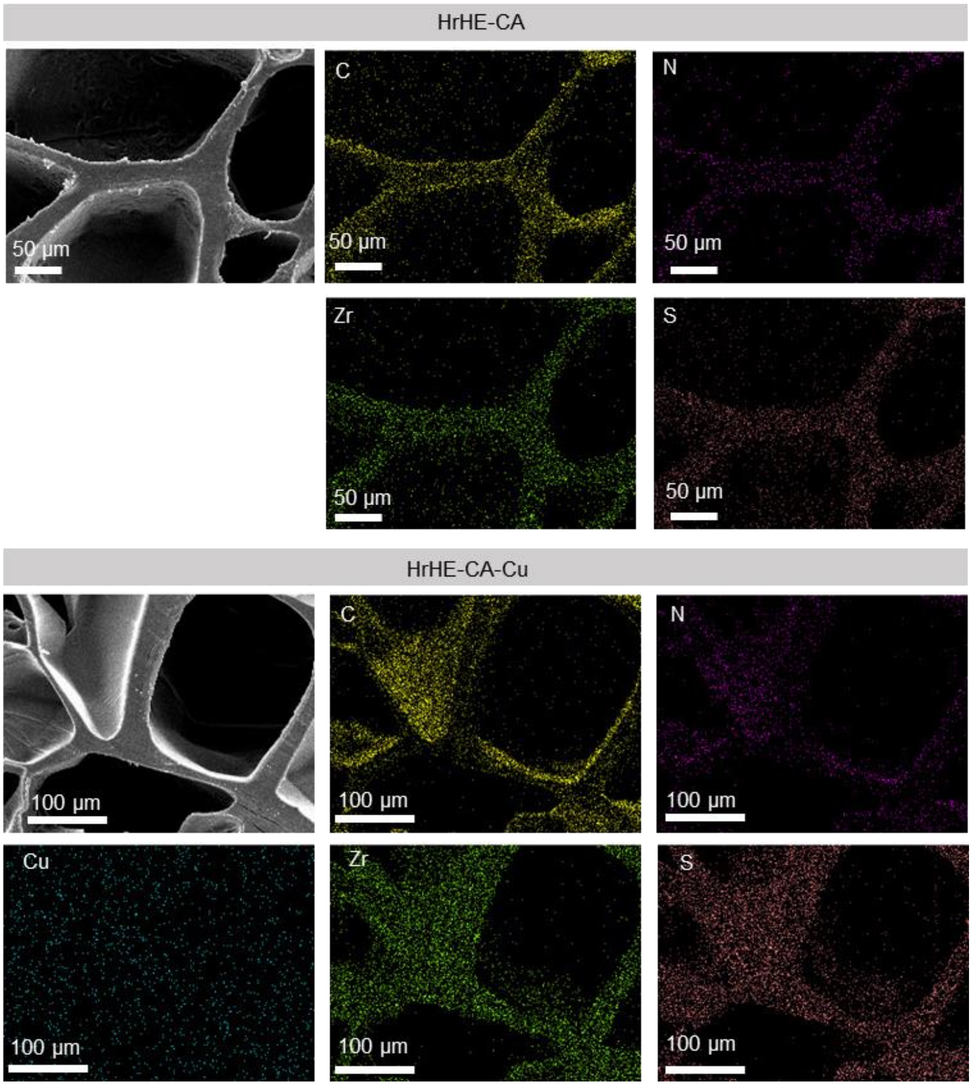

Figure S10. SEM images for HrHE-CA (upper panel) and HrHE-CA-Cu (lower panel), with corresponding energy dispersive X-ray (EDX) elemental mapping.

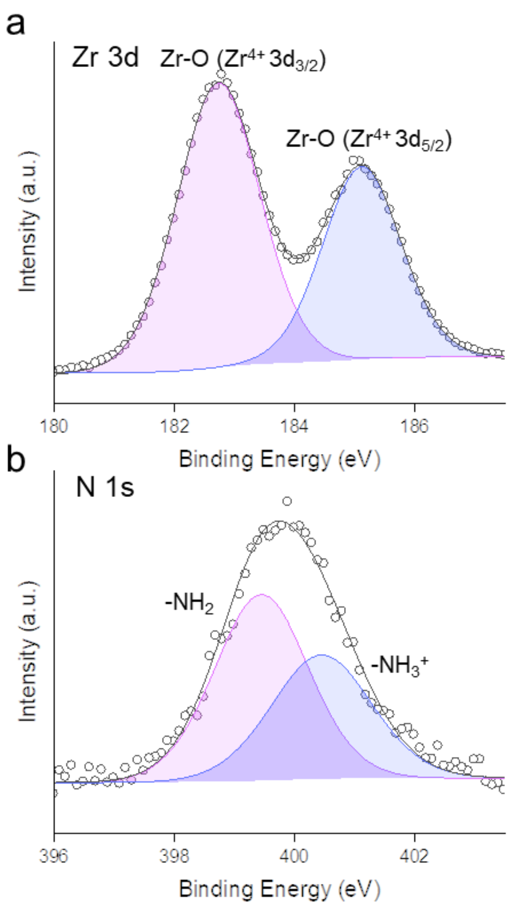

Figure S11. XPS spectra for HrHE-CA. The two peaks in the deconvoluted XPS spectrum of Zr 3d (**a**) at 182.7 and 185.1 eV are attributed to Zr^{4+} $3d_{5/2}$ and $3d_{3/2}$, respectively, suggesting the formation of Zr-O bonds in the metal cluster and the assembly of chromophore. The high-resolution spectrum of N 1s (**b**) shows the contribution of $–NH_2$ bonded to the phenylene of the linker (–NH2, 399.5 eV) and the protonated amidogen form ($–NH_3^+$, 400.5 eV).

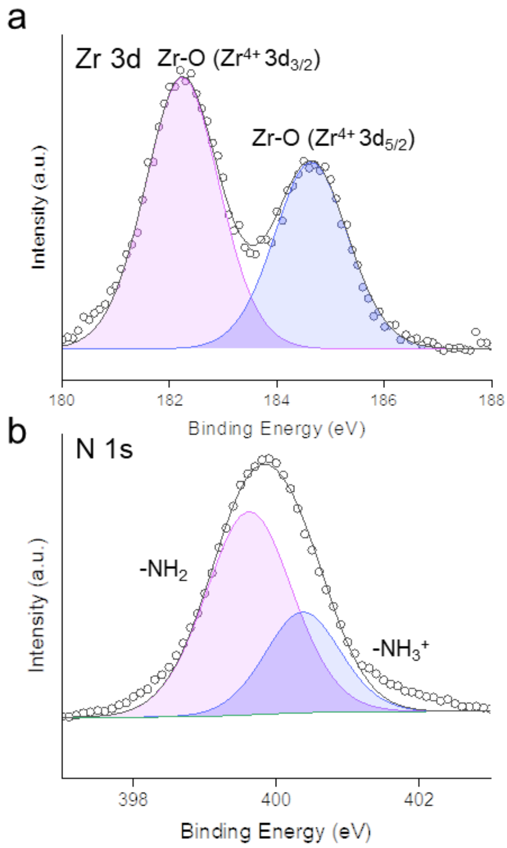

Figure S12. XPS spectra for HrHE-CA-Cu. The two peaks in the XPS spectrum of Zr 3d (**a**) at 182.3 and 184.6 eV are attributed to Zr^{4+} $3d_{5/2}$ and $3d_{3/2}$, respectively, suggesting the deposition of Cu catalysts did not affect the chemical stability of the chromophore assembly. The XPS spectrum of N 1s (**b**) shows the similar peaks like HrHE-CA, $–NH_2$ bonded to the phenylene of the linker (–NH2, 399.6 eV) and the protonated amidogen form ($–NH_3^+$, 400.4 eV).

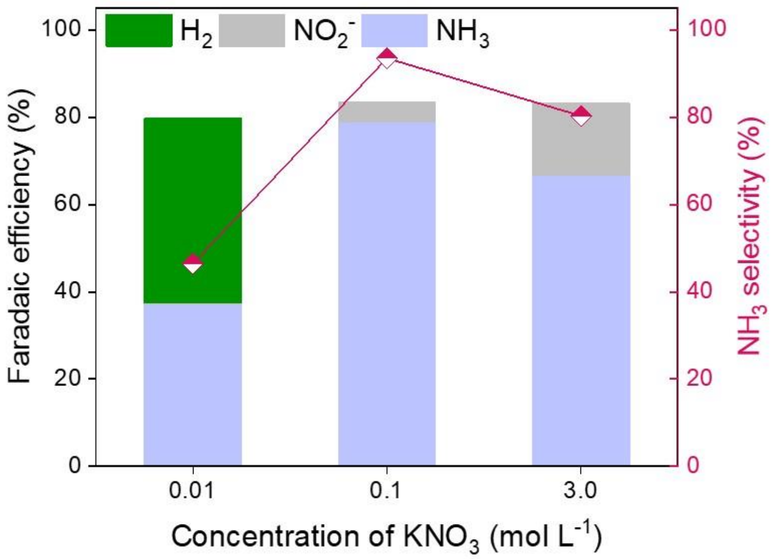

Figure S13. PEC efficiencies for NH_3 production with different concentrations of NO_3^-.

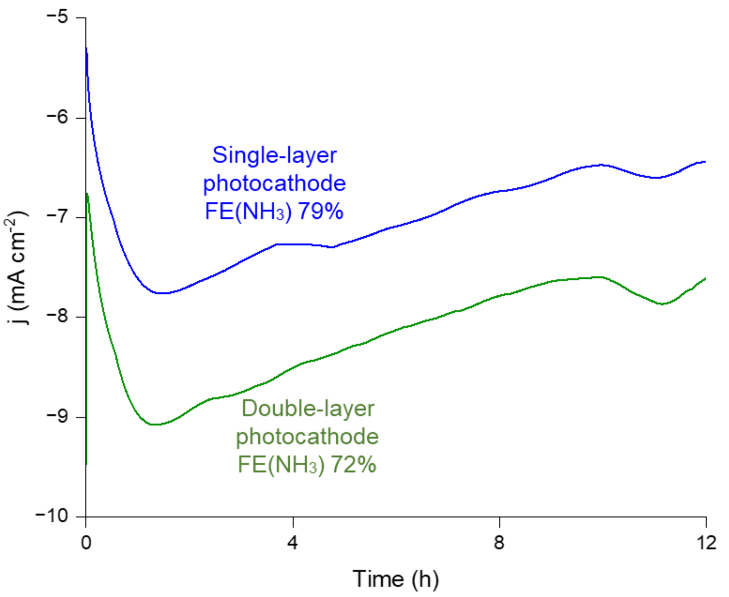

Figure S14. 12-hour photoelectrocatalytic current densities with single-layer and double-layer photocathodes (HrHE-CA-Cu) in the presence of NO_3^- (0.10 M). Solar irradiation intensity: 100 mW cm^{-2} (AM 1.5 G).

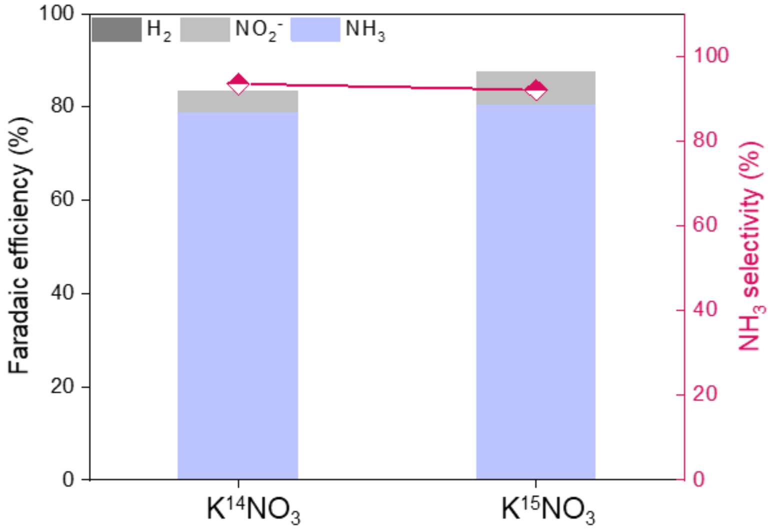

Figure S15. ^{15}N-isotope-labelling experiment for determining the N source for the produced NH_3. From the results, feeding the system with $^{15}NO_3^-$ gives the same results as that with $^{14}NO_3^-$, evincing that the N source for the produced NH_3 is completely from NO_3^- in the electrolyte.

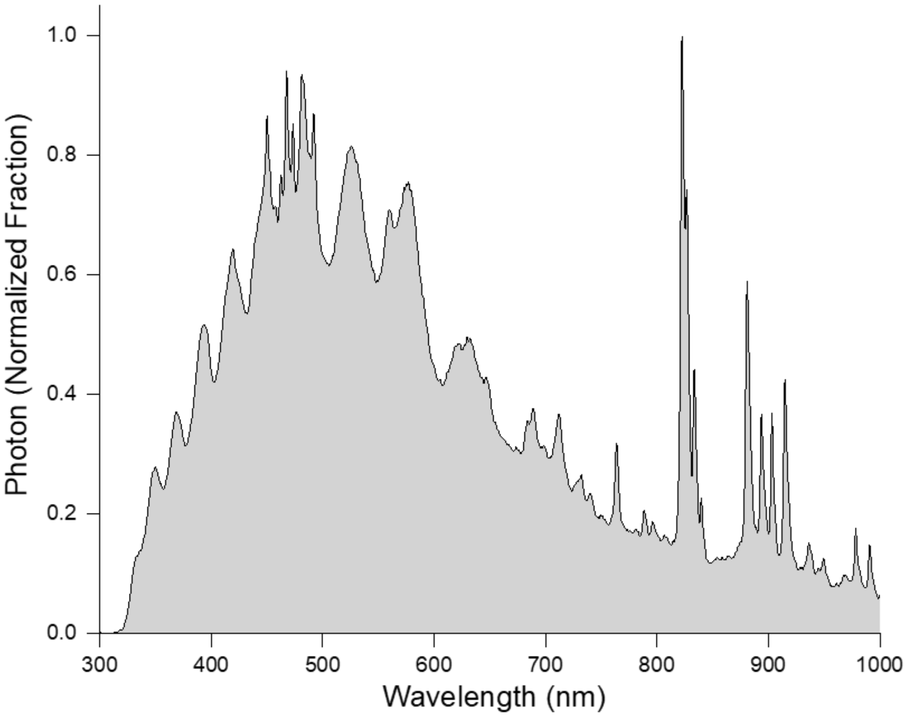

Figure S16. Simulated solar irradiation spectrum (AM 1.5 G) used for the PEC experiments.

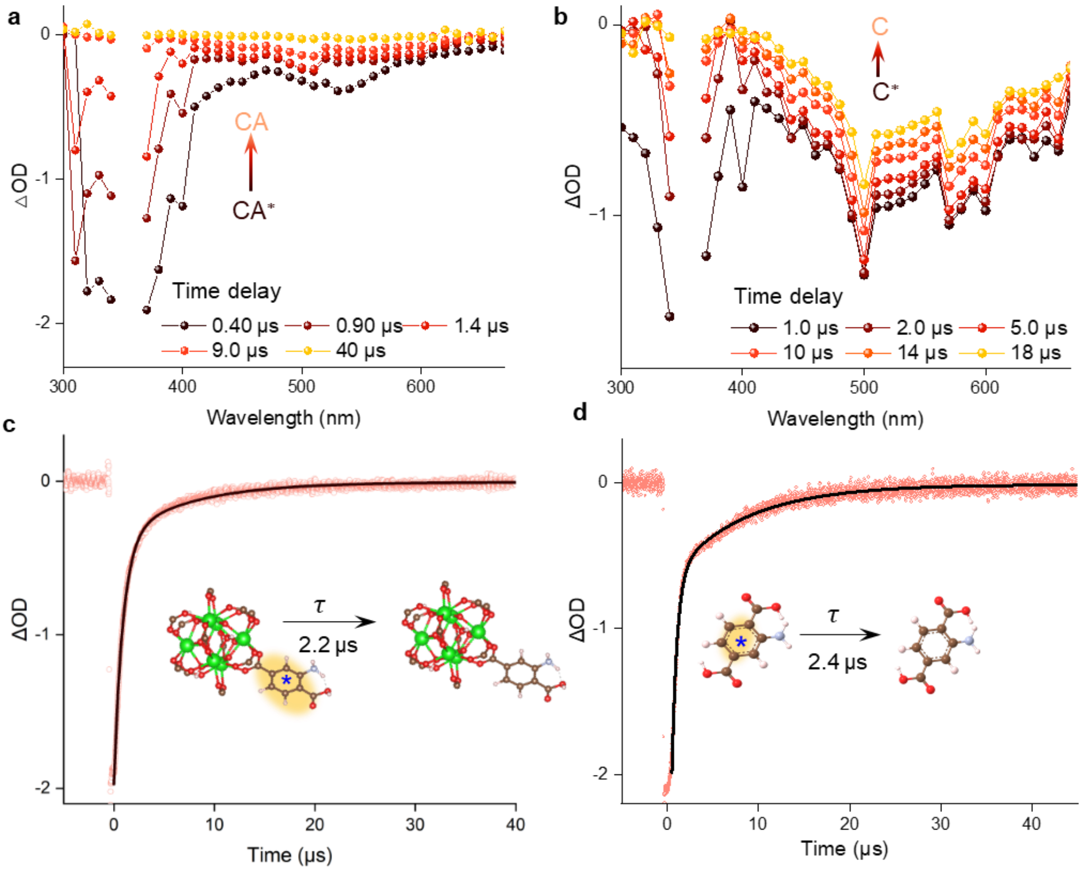

Figure S17. Nanosecond transient absorption (TA) spectra for the chromophores without Cu catalyst. a,b, TA spectra following photoexcitation of CA (zirconium-coordinated 2-aminoterephthalic acid) (**a**) and 2-aminoterephthalic acid (denoted as C) (**b**) at 355 nm, shown as a function of probe delay time. **c,d,** Time-resolved TA traces probed at 370 nm showing the ground state bleach of CA (**c**) and C (**d**). Red dot: experimental data. Black line: fitted data based on biexponential decay function.

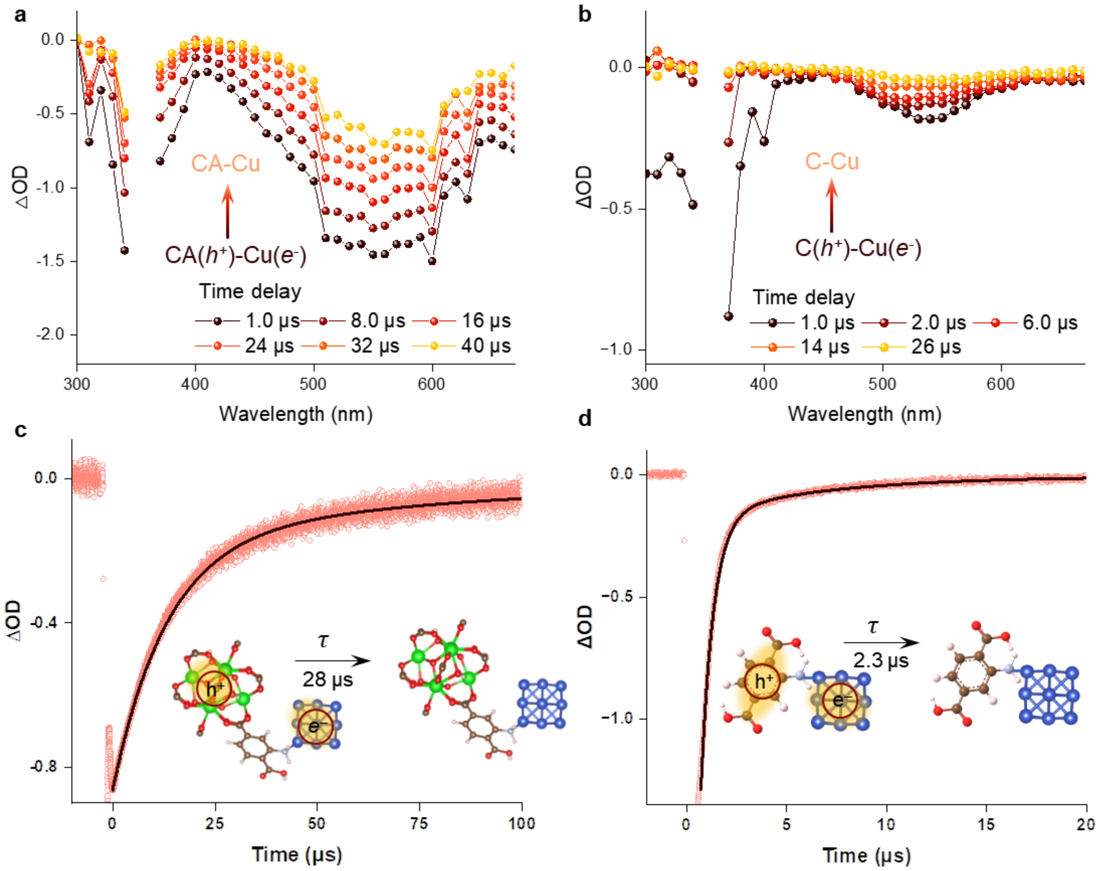

Figure S18. Nanosecond TA spectra for the chromophores with Cu catalyst. a,**b**, TA spectra following photoexcitation of CA-Cu (**a**) and C-Cu (**b**) at 355 nm, shown as a function of probe delay time. **c**,**d**, Time-resolved TA traces probed at 370 nm for CA-Cu (**c**) and C-Cu (**d**). Red dot: experimental data. Black line: biexponential decay fits.

Time-resolved decay kinetics. The biexponential decay model used for fitting the decay kinetics is shown below:

$\Delta OD\ (t) = A_1 exp(-t/\tau_1) + A_2 exp(-t/\tau_2)$

The obtained lifetimes τ_1,τ_2, and corresponding percentages, A_1, A_2, are summarized in Table S8. The weighted average of lifetime (τ) can be calculated by the formula:

$$\tau = \frac{A_1}{A_1+A_2} \times \tau_1 + \frac{A_2}{A_1+A_2} \times \tau_2$$

Quantum yields. The quantum yields were calculated using $Ru(bpy)_3^{2+}$ as a reference standard according to the equation[14]:

$$\frac{\bar{\phi}_s}{\bar{\phi}_{Ru}} = \frac{\left(\frac{\Delta OD_s}{\varepsilon_s}\right)}{F_s} / \frac{\left(\frac{\Delta OD_{Ru}}{\varepsilon_{Ru}}\right)}{F_{Ru}}$$

The transient absorption probed at 450 nm after excitation at 355 nm was used for calculation. $\bar{\phi}$ is the quantum yield, ΔOD is the transient absorption at 450 nm, ε is the molar absorption coefficient at 450 nm, F is the fraction of light absorption at 355 nm calculated by the formula: $F=1-10^{-A}$ (A is the absorbance of the samples). The quantum yields of CA-Cu and C-Cu probed at 2.0 μs, were determined to be 0.87 and 0.033, respectively.

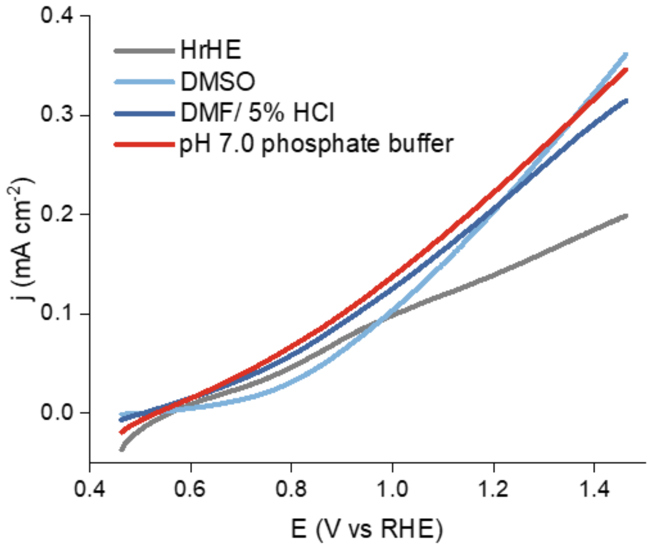

Figure S19. **Linear sweep voltammograms for HrHE heated at 100 °C for 24 hours in DMSO, DMF or pH 7.0 phosphate buffer.** Electrolyte: pH 7.0 phosphate buffer with 30 mM hydroquinone. Scan rate: 5.0 mV s^{-1}.

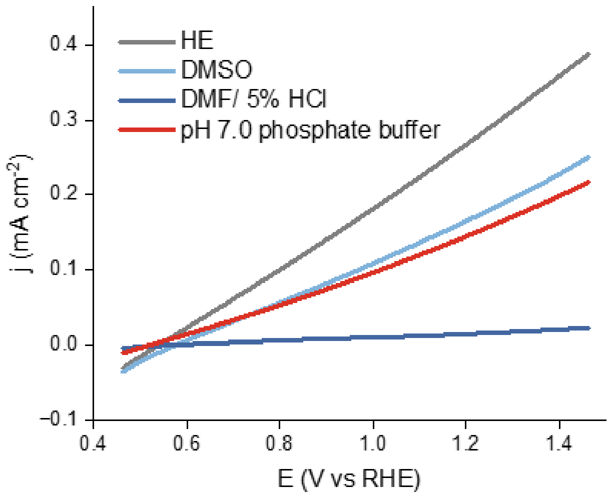

Figure S20. **Linear sweep voltammograms for HE without heat-resistant components after heating at 100 °C for 24 hours in DMSO, DMF with 5% HCl or pH 7.0 phosphate buffer.** Electrolyte: pH 7.0 phosphate buffer with 30 mM hydroquinone. Scan rate: 5.0 mV s^{-1}.

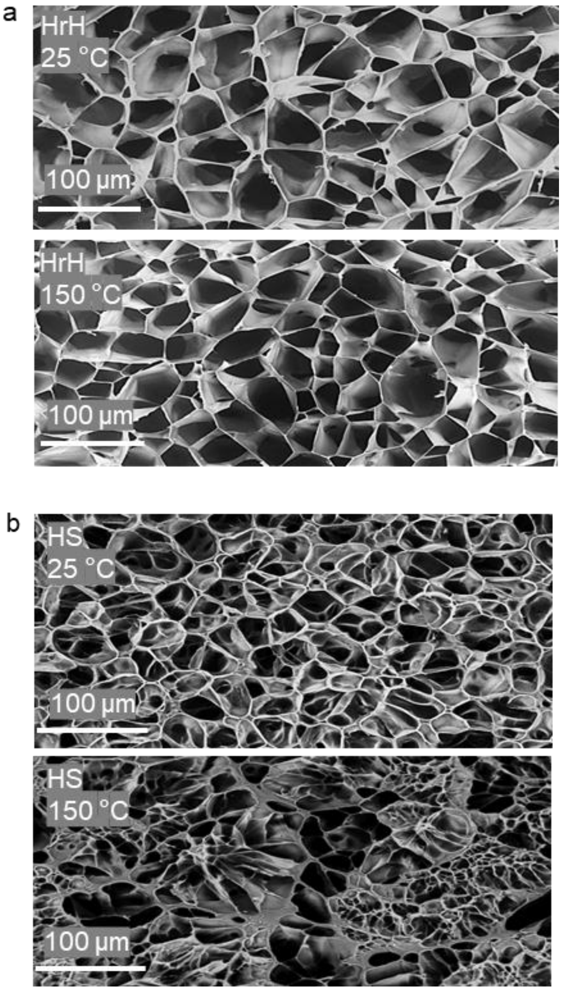

Figure S21. SEM micrographs for the freeze-dried HrH (**a**) and the hydrogel substrate without heat-resistant components (HS) (**b**) under 25 °C (upper panel) and 150 °C for two days (lower panel).

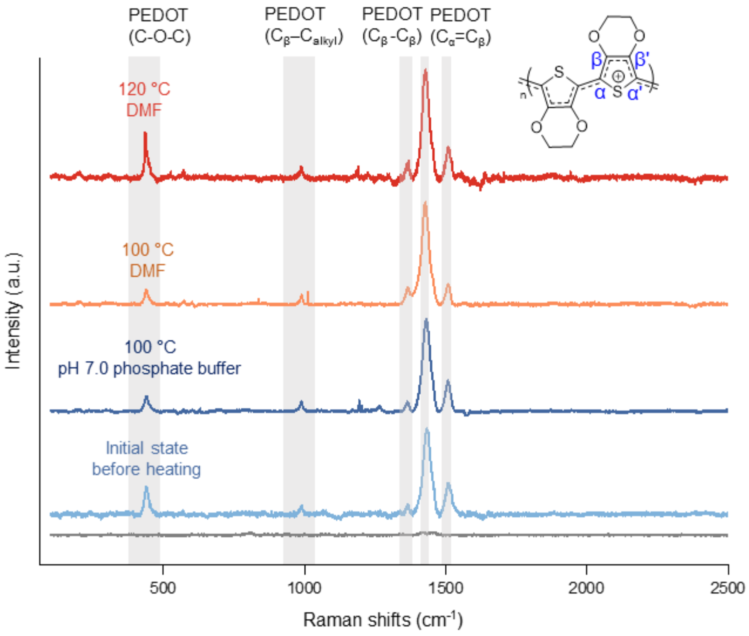

Figure S22. Raman spectra for HrHE before and after heating in DMF and pH 7.0 phosphate buffer for 24 hours. The bottom gray line is the background spectrum for the covalent scaffold (HrH).

In the spectrum for HrHE under room temperature at the initial state before heating (light blue curve), the peak at 1427 cm^{-1} is attributed to the symmetrical C_α=C_β vibration of the thiophene ring.[15-16] The two surrounding peaks at 1365 cm^{-1} and 1508 cm^{-1} correspond to the C_β-C_β inter-ring stretching and asymmetrical C_α=C_β vibrations, respectively. The other peaks observed at 440 cm^{-1} and 987 cm^{-1} are assigned to C-O-C distortion and C_β–C_{alkyl} stretching, respectively. No obvious changes were observed for the samples after heating treatments, which indicates high structural stability of HrHE under elevated temperatures.

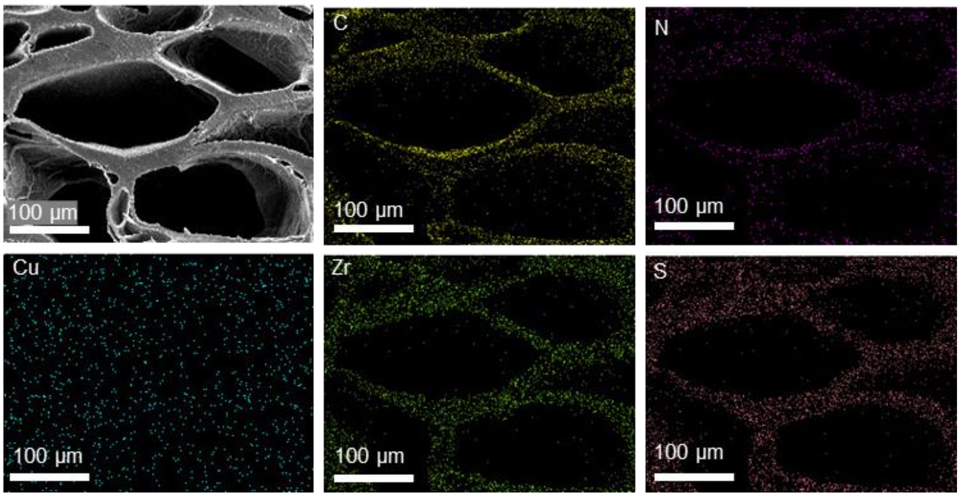

Figure S23. SEM images and corresponding EDX elemental mapping for the photoelectrode after long-term photoelectrocatalysis.

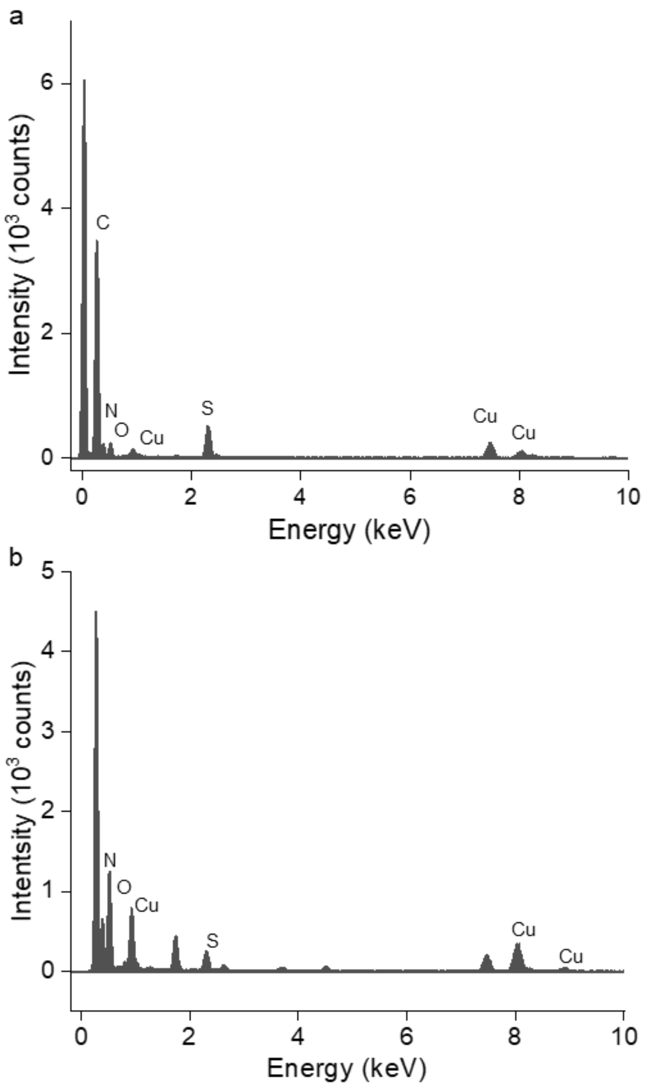

Figure S24. EDX spectra for HrHE-CA-Cu before (a) and after photoelectrocatalysis (b). Electrolyte: KNO_3 (0.10 M) in pH 4.5 acetate buffer (1.0 M) with Na_2SO_4 (0.50 M) as the supporting electrolyte.

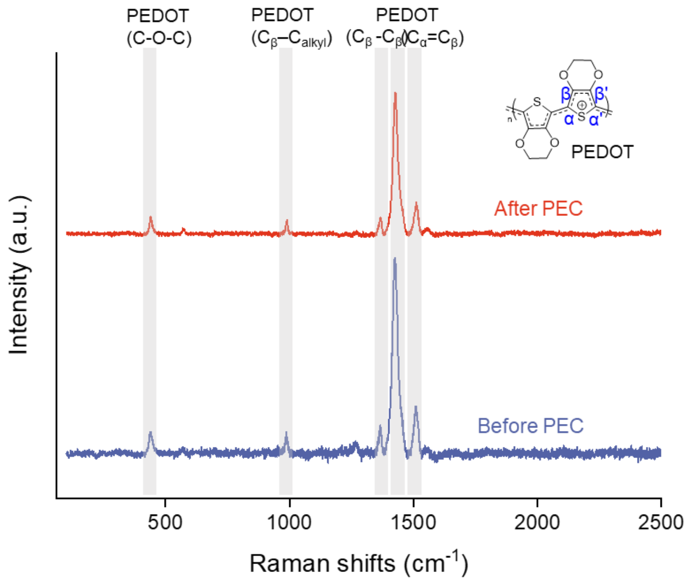

Figure S25. Raman spectra for the photoelectrode, HrHE-CA-Cu, before and after long-term PEC experiment.

The Raman spectrum before PEC experiment (blue line) exhibits features for PEDOT at 441 cm^{-1} (C-O-C distortion), 987 cm^{-1} (C_β–C_{alkyl} stretching), 1367 cm^{-1} (C_β-C_β inter-ring stretching), 1425 cm^{-1} (symmetrical C_α=C_β vibration of the thiophene ring) and 1510 cm^{-1} (asymmetrical C_α=C_β vibrations). After PEC experiment, the above features remain (red line) with negligible changes, indicating high structural stability of the covalent network.

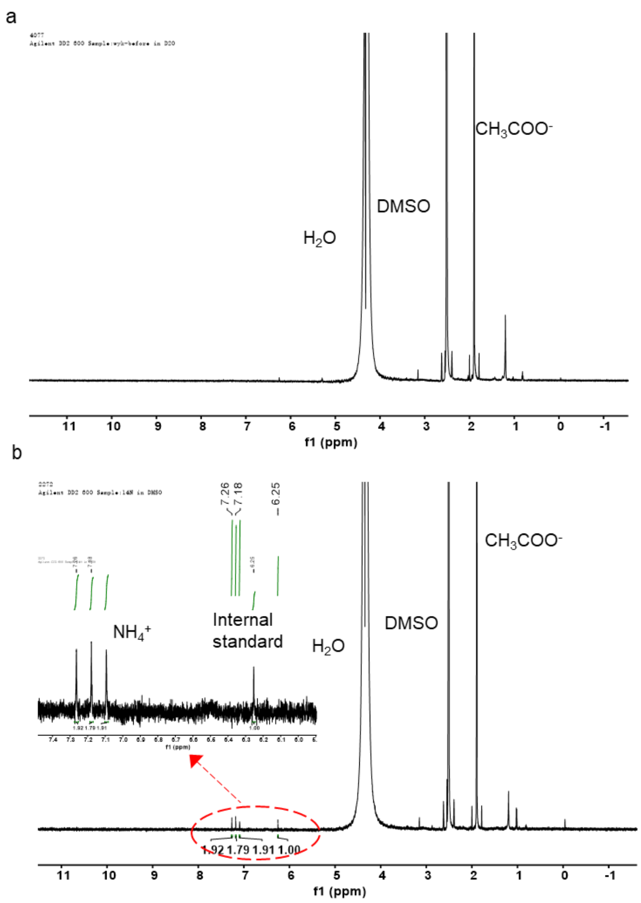

Figure S26. ^{1}H-NMR spectra for electrolytes before (a) and after (b) long-term photoelectrocatalysis.

The ^{1}H NMR spectrum of electrolyte before photoelectrocatalysis displayed characteristic peaks of H_2O (4.31 ppm), DMSO (2.51 ppm), maleic acid (internal standard, 6.25 ppm), CH_3COO^- (from pH buffer, 1.90 ppm), and a few grease impurities (1.20 ppm, 0.80 ppm). After photoelectrocatalysis, similar peaks were observed and a triplet peak of NH_4^+ at 7.18 ppm. Except the above features, no signals for new organic species are observed.

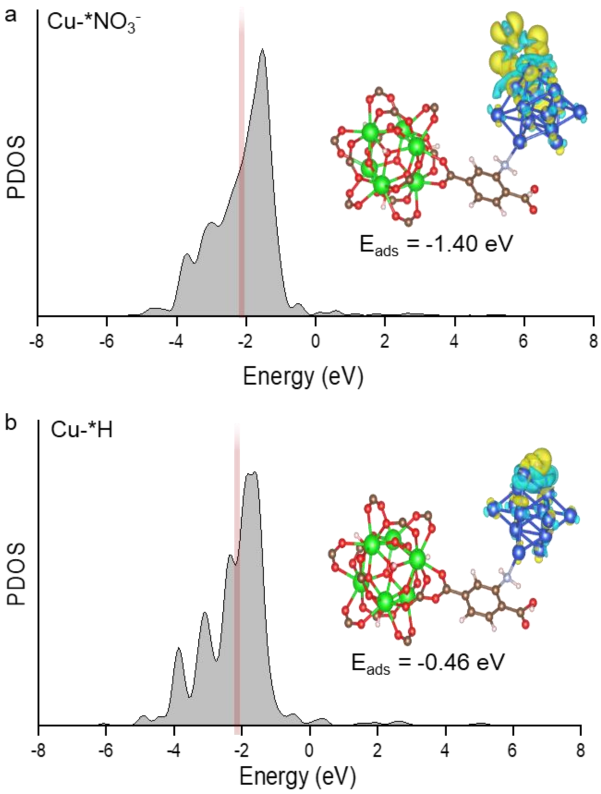

Figure S27. Projected density of states (PDOS) for adsorbed $*NO_3^-$ (a) and *H (b) on CA-Cu with the corresponding charge density difference configurations. Blue, red, green, brown, pink and silver spheres represent Cu, O, Zr, C, H and N atoms, respectively. Cyan and yellow regions indicate electron-donating and electron-withdrawing areas, respectively. The d-band centers for $*NO_3^-$ and *H adsorbed Cu are analyzed to be -2.04 and -2.16, respectively.

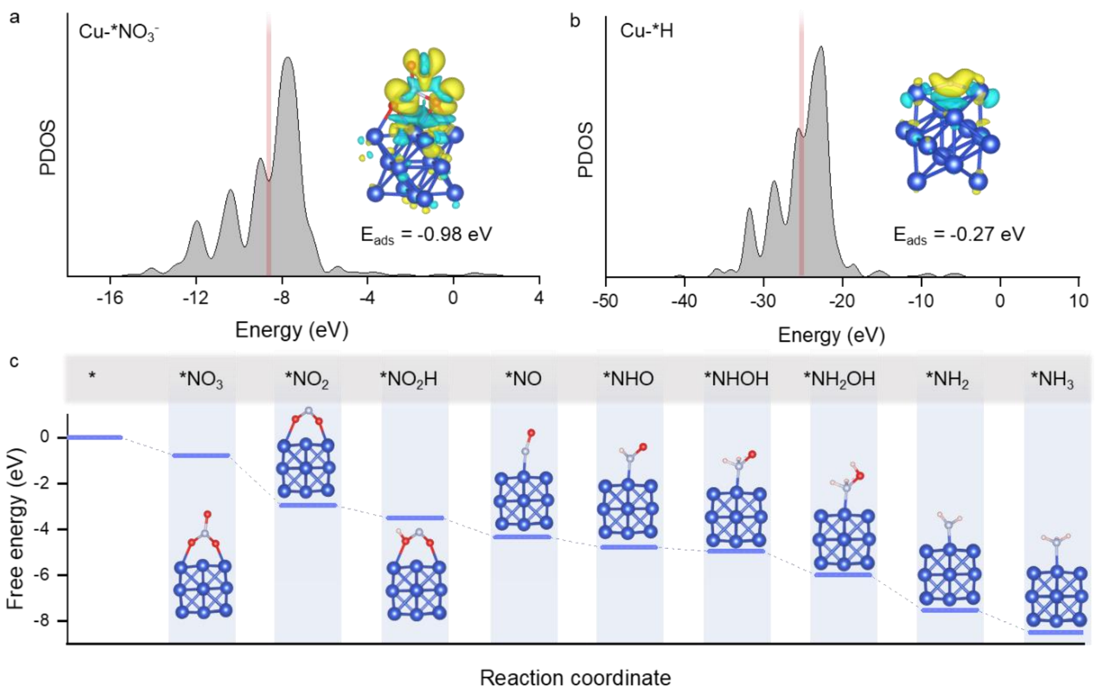

Figure S28. PDOS for adsorbed *NO_3^- (a) and *H (b) on Cu catalysts with the corresponding charge density difference configurations. Energy barriers for the catalytic intermediates during NO_3^- reduction to NH_3 on Cu catalysts (c). Blue, red, pink and silver spheres represent Cu, O, H and N atoms, respectively. Cyan and yellow regions indicate electron-donating and electron-withdrawing areas, respectively. The d-band centers for *NO_3^- and *H adsorbed Cu are analyzed to be -8.64 and -25.0 eV, respectively.

Table S1. Summaries of contact angles, pore sizes and porosities for HrH, HrHE, HrHE-CA and HrHE-CA-Cu.

Sample	Contact angle	Pore size /μm	Porosity%
HrH	16.5°	74 - 159	95.0
HrHE	42.3°	67 - 112	89.0
HrHE-CA	46.5°	58 - 110	86.9
HrHE-CA-Cu	46.1°	60 - 115	84.8
HrHE-CA-Cu (After long-term PEC experiment)	47.5°	67 - 120	85.8

Table S2. Band edge potentials of CA and HrHE.

Sample	E_{CB} /V vs NHE	E_{VB} /V vs NHE	E_g /eV
HrHE	-0.8	0.59	1.39
CA	–0.37 [17]	2.46 [17]	2.83 [17]

E_{CB}: conduction band edge potential.
E_{VB}: valence band edge potential.
E_g : bandgap energy.

Table S3. Surface coverages of the components in the CA photocathode.

	Surface coverage /mM
CA<br>(per unit cell)	11[a]
Zr	67[b]
H_2ATA	67[c]
Cu	32[b]

[a] The surface coverage of CA is estimated by approximation based on cell units and the amount of Zr.

[b] The surface coverages of Zr and Cu are estimated by inductively coupled plasma optical emission spectroscopy (ICP-OES).

[c] The surface coverage of H_2ATA is estimated by the ratio of H_2ATA to Zr in a CA unit cell.

Table S4. Summary of photoelectrocatalytic efficiencies for the photocathode at pH 4.5.

E_{app} (V vs RHE)	Solar-to-electron efficiency% [a]	Photogenerated electron-to-NH_3 efficiency% [b]	Solar-to-NH_3 efficiency% [c]
-0.30	19	76	14
-0.20	7.7	77	5.9
-0.10	1.9	78	1.5
0.10	0.10	73	0.073
0.30	0.050	49	0.025

[a] Solar-to-electron efficiency% = $\frac{\text{n(photogenerated electrons)}}{\text{n (photons)}}$. The n(photogenerated electrons) is quantified based on photocurrent densities.

[b] Photogenerated electron-to-NH_3 efficiency% = $8\times\frac{n(NH_3) - n(NH_3)_{Dark}}{\text{n(photogenerated electrons)}}$.

[c] Solar-to-NH_3 efficiency% = $8\times\frac{n(NH_3) - n(NH_3)_{Dark}}{\text{n(photons)}}$. The $n(NH_3)_{Dark}$ is the amount of NH_3 produced under dark.

Table S5. Comparisons on solar-to-NH_3 performances.

Photocathode	pH	$[NO_3^-]$ /M	Irradiation intensity /mW cm^{-2}	Current density /mA cm^{-2}	Yield rate [a] /μmol h^{-1} cm^{-2}	Applied bias /V vs RHE	FE% [b]	Reference
HrHE-CA-Cu	4.5	0.10	100	7.1	28	-0.30	79	This work
$CuPc/CeO_2$	6.9	0.10	NA (300 W Xenon lamp)	0.75	1.2	-0.60	33	[18]
$ZnIn_2S_4/BiVO_4$	7.0	0.0024	100	3.8	1.8	-0.10	37	[19]
CeO_2-C/$BiVO_4$	7.0	0.0016	100	0.50	1.3	-0.10	32	[20]
NiO/Au plasmon/TiO_2	7.0	0.20	44 (532 nm)	1.4	3.0	-0.60	44	[21]
O_SiNW/Au	7.0	0.010	100	0.060	0.26	0.20	96	[22]
Fe-MoS_2	13	0.10	100	5.5	25	-0.48	98	[23]
$BiVO_4$/CuPc	7.0	0.0016	100	0.80	0.76	0.10	20	[24]

[a] Yield rate of NH_3 production.

[b] Faradaic efficiency (FE) of NH_3 production.

Table S6. 12-hour photoelectrocatalytic performances of single- or double-layer photocathodes, HrHE-CA-Cu, under various conditions including nitrate concentrations and solar irradiation intensities. Electrolyte: argon-degassed KNO_3 in pH 4.5 acetate buffer (1.0 M).

HrHE-CA-Cu samples	Conditions		Photoelectrocatalytic performances				
	$[NO_3^-]$ / M	Irradiation / Sun (0.1 W/cm^2)	j /mA cm^{-2}	n(NH_3) /µmol cm^{-2}	FE% [a]	Selectivity%	EQE% [b]
Single-layer	0.10	1.0	7.1	336	79	92	14
Single-layer	3.0	1.0	7.5	169	36	64	7.5
Double-layer	0.10	1.0	8.1	325	72	96	14
Single-layer	0.10	4.0	8.3	345	74	93	3.8

[a] Faradaic efficiency (FE) of NH_3 production.

[b] External quantum efficiency (EQE) of NH_3 production based on solar photon flux.

Table S7. Ionic-limiting factors that influence the PEC NO_3^--to-NH_3 efficiencies.

PEC Product / Electrolyte	limiting factor	$FE(NH_3)$ %	$FE(NO_2^-)$ %	$FE(H_2)$ %	NH_3 selectivity %
0.10 M acetate buffer with 0.50 M Na_2SO_4	buffer concentration	51	47	0.0	52
1.0 M acetate buffer	ionic strength	96	11	0.0	89
1.0 M acetate buffer with 0.50 M Na_2SO_4	best performing	86	9.3	0.0	90

Table S8. TA Fitting parameters determined from fitting of the kinetic TA traces for CA, C, CA-Cu and C-Cu in Figures S16 and S17 with biexponential decay function.

Sample	$\tau 1$ (μs)	$\frac{A_1}{A_1 + A_2}$ %	τ_2 (μs)	$\frac{A_2}{A_1 + A_2}$ %	τ (μs) [1]
CA	0.91	80	7.2	20	2.2
C	0.57	75	0.63	25	2.4
CA-Cu	13	81	91	19	28
C-Cu	0.63	71	6.2	29	2.3

[1] τ denotes weighted average of lifetime by $\tau = \frac{A_1}{A_1+A_2} \times \tau_1 + \frac{A_2}{A_1+A_2} \times \tau_2$.

Table S9. The percentage of metal (Cu and Zr) leakage from the photoelectrode to electrolyte during long-term photoelectrocatalysis examined by ICP-OES.

	Cu %	Zr %
Percentage of metal leakage to the electrolyte	4.0	0.0

References

[1] B. Salunkhe, T. Schuman, A. Al Brahim, B. Bai, *Chem. Eng. J.* **2021,** *426*.

[2] Z. Y. Wu, M. Karamad, X. Yong, Q. Huang, D. A. Cullen, P. Zhu, C. Xia, Q. Xiao, M. Shakouri, F. Y. Chen, J. Y. T. Kim, Y. Xia, K. Heck, Y. Hu, M. S. Wong, Q. Li, I. Gates, S. Siahrostami, H. Wang, *Nat. Commun.* **2021,** *12*, 2870.

[3] R. Y. Hodgetts, A. S. Kiryutin, P. Nichols, H.-L. Du, J. M. Bakker, D. R. Macfarlane, A. N. Simonov, *ACS Energy Lett.* **2020,** *5*, 736.

[4] X. Huang, L. Yang, R. Emanuelsson, J. Bergquist, M. Stromme, M. Sjodin, A. Gogoll, *Beilstein J. Org. Chem.* **2016,** *12*, 2682.

[5] S. Zhang, M. Li, J. Li, Q. Song, X. Liu, *Proc. Natl. Acad. Sci. U.S.A.* **2022,** *119*.

[6] G. Kresse, J. Furthmüller, *Comput. Mater. Sci.* **1996,** *6*, 15.

[7] J. P. Perdew, K. Burke, M. Ernzerhof, *Phys. Rev. Lett.* **1996,** *77*, 3865.

[8] P. E. Blochl, *Phys. Rev. B* **1994,** *50*, 17953.

[9] S. Grimme, *J. Comput. Chem.* **2006,** *27*, 1787.

[10] A. Kausar, *Int. J. Polymer. Mater.* **2015,** *64*, 184.

[11] G. Su, S. Yin, Y. Guo, F. Zhao, Q. Guo, X. Zhang, T. Zhou, G. Yu, *Mater. Horizons* **2021,** *8*, 1795.

[12] J. M. Kurth, C. Dahl, J. N. Butt, *J. Am. Chem. Soc.* **2015,** *137*, 13232.

[13] M. Peñas-Garzón, M. J. Sampaio, Y. L. Wang, J. Bedia, J. J. Rodriguez, C. Belver, C. G. Silva, J. L. Faria, *Sep. Purif. Technol.* **2022,** *286*.

[14] B. Shan, S. Vanka, T.-T. Li, L. Troian-Gautier, M. K. Brennaman, Z. Mi, T. J. Meyer, *Nat. Energy* **2019,** *4*, 290.

[15] J. Atoyo, M. R. Burton, J. McGettrick, M. J. Carnie, *Polymers* **2020**, *12*, 559.

[16] S. V. Selvaganesh, J. Mathiyarasu, K. L. N. Phani, V. Yegnaraman, *Nanoscale Res. Lett.* **2007,** *2*.

[17] Y. Wang, Q. Yang, F. Yi, R. Lu, Y. Chen, C. Liu, X. Li, C. Wang, H. Yan, *ACS Appl. Mater. Interfaces* **2021,** *13*, 29916.

[18] X. Li, W. Fan, Y. Bai, Y. Liu, F. Wang, H. Bai, W. Shi, *Chem. Eng. J.* **2022,** *433*, 133225.

[19] F. Wang, Q. Ding, J. Ding, Y. Bai, H. Bai, W. Fan, *Chem. Eng. J.* **2022,** *450*, 138260.

[20] H. Bai, F. Wang, Q. Ding, W. Xie, H. Li, G. Zheng, W. Fan, *Inorganic Chemistry* **2023,** *62*, 2394.

[21] V. R. Silveira, R. Bericat-Vadell, J. Sá, *J. Phys. Chem. C* **2023,** *127*, 5425.

[22] H. E. Kim, J. Kim, E. C. Ra, H. Zhang, Y. J. Jang, J. S. Lee, *Angew. Chem. Int. Ed.* **2022,** *61*, e202204117.

[23] J. Li, Y. Zhang, C. Liu, L. Zheng, E. Petit, K. Qi, Y. Zhang, H. Wu, W. Wang, A. Tiberj, X. Wang, M. Chhowalla, L. Lajaunie, R. Yu, D. Voiry, *Adv. Funct. Mater.* **2022,** *32*, 2108316.

[24] Y. Bai, S. Gao, W. Xie, Z. Fang, H. Bai, W. Fan, *Int. J. Hydrog. Energy* **2023,** *48*, 10882.
